# Mapping the abundance of endemic mosquito-borne diseases vectors in southern Quebec

**DOI:** 10.1186/s12889-023-15773-x

**Published:** 2023-05-22

**Authors:** Antoinette Ludwig, François Rousseu, Serge Olivier Kotchi, Julie Allostry, Richard A. Fournier

**Affiliations:** 1 Public Health Risk Sciences Division, National Microbiology Laboratory, Public Health Agency of Canada, 3200 Rue Sicotte, Saint-Hyacinthe, QC J2S 2M2 Canada; 2 Groupe de recherche en épidémiologie des zoonoses et santé publique, Faculty of Veterinary Medicine, Université de Montréal, 3200 Rue Sicotte, Saint Hyacinthe, QC J2S 2M2 Canada; 3 Department of Biology/Centre d’étude de la forêt, Université de Sherbrooke, Sherbrooke, QC J1K 2R1 Canada; 4 GéoMont - Agence géomatique montérégienne, 166, rue Cowie, suite 105, Granby, QC J2G 3V3 Canada; 5 Department of Applied Geomatics, Centre d’Applications et de Recherches en Télédétection, Université de Sherbrooke, Sherbrooke, QC J1K 2R1 Canada

**Keywords:** Public health, Mosquitoes, Zoonoses, Vector-borne diseases, Space-time modelling, Southern Quebec, *Culex pipiens-restuans*, *Aedes Ochlerotatus stimulans*, *Coquillettidia perturbans*, *Aedes vexans*

## Abstract

**Background:**

Climate change is increasing the dispersion of mosquitoes and the spread of viruses of which some mosquitoes are the main vectors. In Quebec, the surveillance and management of endemic mosquito-borne diseases, such as West Nile virus or Eastern equine encephalitis, could be improved by mapping the areas of risk supporting vector populations. However, there is currently no active tool tailored to Quebec that can predict mosquito population abundances, and we propose, with this work, to help fill this gap.

**Methods:**

Four species of mosquitos were studied in this project for the period from 2003 to 2016 for the southern part of the province of Quebec: *Aedes vexans* (VEX), *Coquillettidia perturbans* (CQP), *Culex pipiens-restuans* group (CPR) and *Ochlerotatus stimulans* group (SMG) species. We used a negative binomial regression approach, including a spatial component, to model the abundances of each species or species group as a function of meteorological and land-cover variables. We tested several sets of variables combination, regional and local scale variables for landcover and different lag period for the day of capture for weather variables, to finally select one best model for each species.

**Results:**

Models selected showed the importance of the spatial component, independently of the environmental variables, at the larger spatial scale. In these models, the most important land-cover predictors that favored CQP and VEX were ‘forest’, and ‘agriculture’ (for VEX only). Land-cover ‘urban’ had negative impact on SMG and CQP. The weather conditions on the trapping day and previous weather conditions summarized over 30 or 90 days were preferred over a shorter period of seven days, suggesting current and long-term previous weather conditions effects on mosquito abundance.

**Conclusions:**

The strength of the spatial component highlights the difficulties in modelling the abundance of mosquito species and the model selection shows the importance of selecting the right environmental predictors, especially when choosing the temporal and spatial scale of these variables. Climate and landscape variables were important for each species or species group, suggesting it is possible to consider their use in predicting long-term spatial variationsin the abundance of mosquitoes potentially harmful to public health in southern Quebec.

**Supplementary Information:**

The online version contains supplementary material available at 10.1186/s12889-023-15773-x.

## Background

In Canada, the arbovirus most commonly transmitted to humans by mosquitoes are West Nile virus (WNV), California serogroup (CSG) viruses, and, less frequently, Eastern equine encephalitis virus (EEEV) [1–7]. Quebec is one of the provinces that is most concerned by the transmission of these viruses. Since 2002, the number of human cases of WNV has varied considerably from year to year, with an average of 27 cases by year and some particularly extreme year like 2018 with 201 confirmed cases, including 15 deaths [8]. No human cases of infection by EEEV have been observed so far, but some positive horses and emus have been observed sporadically, mainly in the Laurentides region, north of Montréal [9]. The number of human cases caused by the CSG viruses is hard to compare between years because cases with any kind of symptoms have been notifiable only since 2019 (before that date, only encephalitis cases were notifiable). Following enhanced surveillance in 2017, 82 cases were detected in Quebec, whereas in 2018 and 2019, without any enhanced surveillance, only 17 and 18 cases respectively were reported [10]. In Quebec, CSG viruses, EEEV and WNV are on a watch list for health professionals, and are all notifiable diseases [8–10]. Better characterization of the risk associated with mosquitoes likely to transmit diseases to humans is therefore a public health issue for Canada in general, and Quebec in particular.

One tool helping public health assess the situation and for decision-making is the threat exposure risk map, or, in this case, the spatial distribution of the mosquito population [11]. It allows for optimization of control (larviciding) or prevention (message to the public) programs. Modelling mosquito abundances is one way to predictively map exposure risks [12–17]. The variables employed for such a mapping often involve the use of meteorological data, such as temperature and precipitation [12, 14, 15, 18–25] and land-cover data, because the populations being modelled are arthropods, organisms that are highly dependent on their environment and the prevailing weather conditions. The environmental data used as explanatory variables vary widely across studies and cover the following major themes: land cover and land use, vegetation indices, hydrographic indices, drainage indices, wetlands, slope and altitudes [18, 20, 24–28].

In Quebec, *Culex pipiens-restuans* (CPR) is the main vector species for WNV [29]. It is also suspected to have some vector competence for EEEV [30, 31]. CPR preferences in terms of habitat have been studied in several regions comparable with our study region. According to studies conducted in Illinois and New York state, CPR is found in woodlands in particular and, to a lesser extent, in fields, grasslands and urban areas, laying their eggs in artificial and natural containers [20, 26, 32, 33]. More recent studies performed in Ontario and southern Quebec highlighted the importance of urban dwellings, proximity of suburban backyards and edge vegetation and mixed or paved areas for the bioecology of CPR [17, 34, 35]. Similarly, favorable climatic conditions for CPR have been discussed in several studies. The climate variables used vary from one study to another, but in general, CPR abundance is associated with lagged variables, representing a mean or cumulative effect over several days prior to the day of capture, of temperature and precipitation variables [20, 23, 36, 37]. As an example, in southern Quebec, CPR abundance was associated with the average over 22 days of the degree days (Celsius degree over 9 °C only, developmental threshold for CPR) before mosquito capture and mean precipitation over 71 days before capture [22].

*Aedes vexans* (VEX), which is very abundant in southern Quebec, has some vector competence for WNV [38–40], EEEV [30], SSHV [39] and JCV [39, 41]. It spawns primarily in the temporary or more permanent aquatic areas that form in agricultural and wetlands areas, in proximity to urbanized landscapes [17, 34, 42, 43]. Other sources indicate that VEX prefer to develop in pools, shallow pasture ponds, woodlands and grasslands [44]. As with CPR, we observe that VEX abundance is associated with both temperature and precipitation lag variables [22, 36, 37].

*Coquillettidia perturbans* (CQP) larvae require aquatic plants, such as reeds or water lilies; they get oxygen from them by piercing their roots. It is therefore a species that requires permanent bogs or marshes for the larval stage of its life cycle [42, 45]. However, while CQP is very common in wetlands and ‘forest’s [46, 47], not much more is known about the other environmental conditions necessary for their development, other than the importance of precipitation [36]. CQP has some known vector competence for EEEV [30] primarily and for WNV [39] and JCV [41, 48] anecdotally.

The environmental preferences of the Oc. stimulans group (SMG) are poorly documented. It seems that SMG prefers to spawn in vernal pools, especially in woodlands [17, 42, 49]. The group is identified as a member of the “spring aedes/ochlerotatus” group and is therefore very abundant in spring [17]. *Ochlerotatus stimulans* (Oc. stimulans), included in the Oc. stimulans group (SMG), has known vector competence for JCV [48] and SSHV [50]. The SMG also include some rarer species such as Oc. fitchii and Oc. riparius. Nothing is reported in literature about their vector competence for JCV, SSHV, WNV or EEEV.

Not all of these mosquito species have the same preferences in terms of larval habitat and climate sensitivity. It is therefore essential that the models developed are species-specific and combine spatial and temporal aspects [12, 14, 25, 51].

In the present work, we study the variations of the spatiotemporal daily abundance of CPR, SMG, CQP and VEX mosquitoes as a function of land-cover and weather variables in southern Quebec by developing of series of spatio-temporal regression models. The purpose of these models was to map the daily mean mosquito abundance, for each of the four species, to guide campaigns to prevent and control the spread of the diseases borne by the selected species.

## Methods

### Study area

**Fig. 1:**
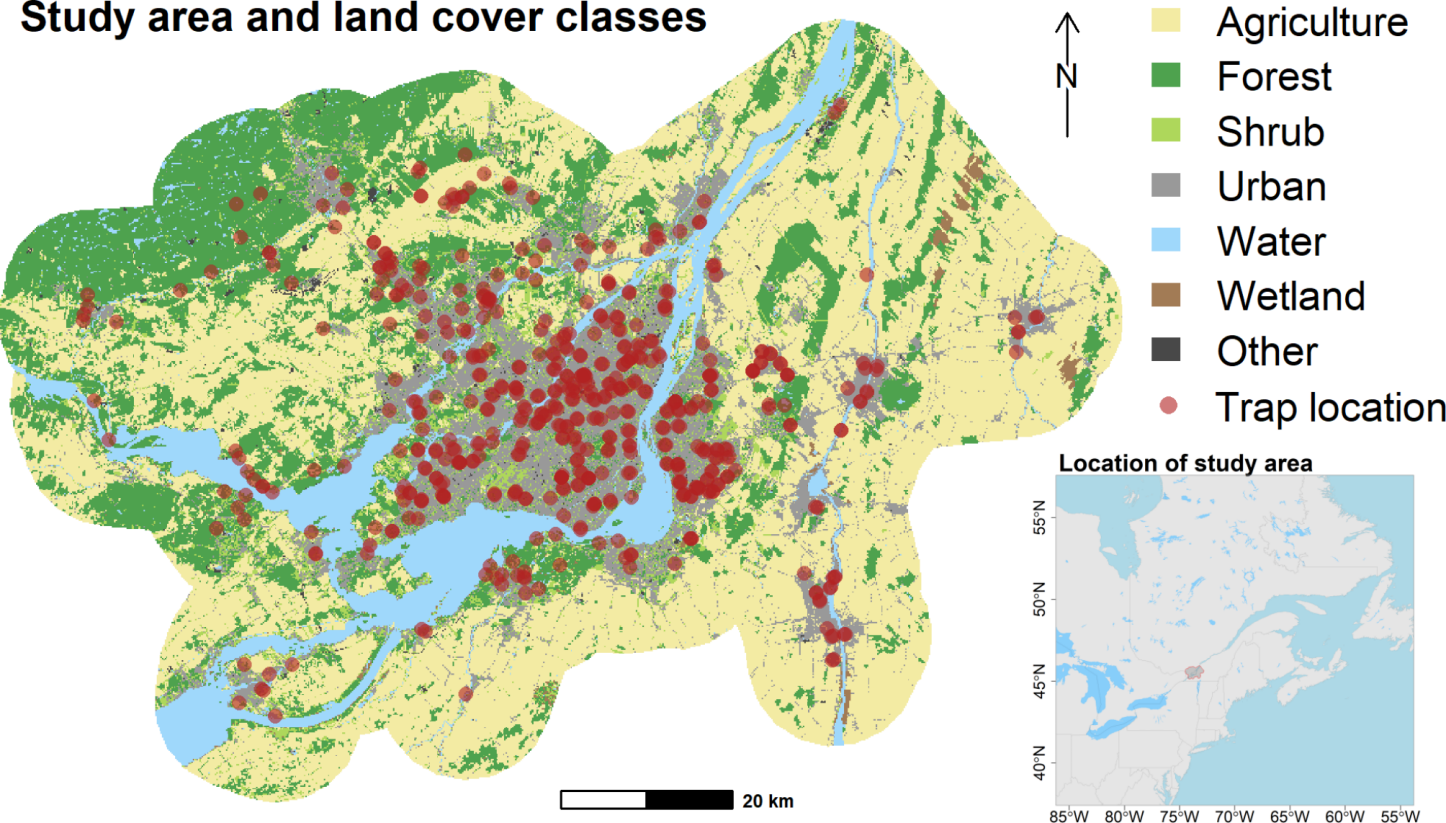
Study area, land-cover classes and mosquito trap locations deployed across the Greater Montréal area, Quebec, Canada (2003–2016)

The study area is located in southern Quebec (Fig. 1) and follows the human ecumene of Greater Montréal. The study area is crossed from southwest to northeast by the St. Lawrence River. Most of the areas south of the river are dense agricultural areas with some open woodlands and the odd wetland. North of the river, past a narrow agricultural strip, there is a ‘forest’ area populated by mixed and coniferous forest whose density increases toward the north. According to the Köppen-Geiger classification [52], most of our study area has a warm-summer humid continental climate (Dfb boreal climate), with the warmest month of the year (July) averaging below 22 °C, but more than four months averaging above 10 °C. The temperature in the coldest four months varies between − 38 °C and 0 °C. Precipitation is distributed evenly throughout the year. In these types of climates, the season of mosquito activity generally extends from May to September.

### Mosquito abundance data

**Fig. 2:**
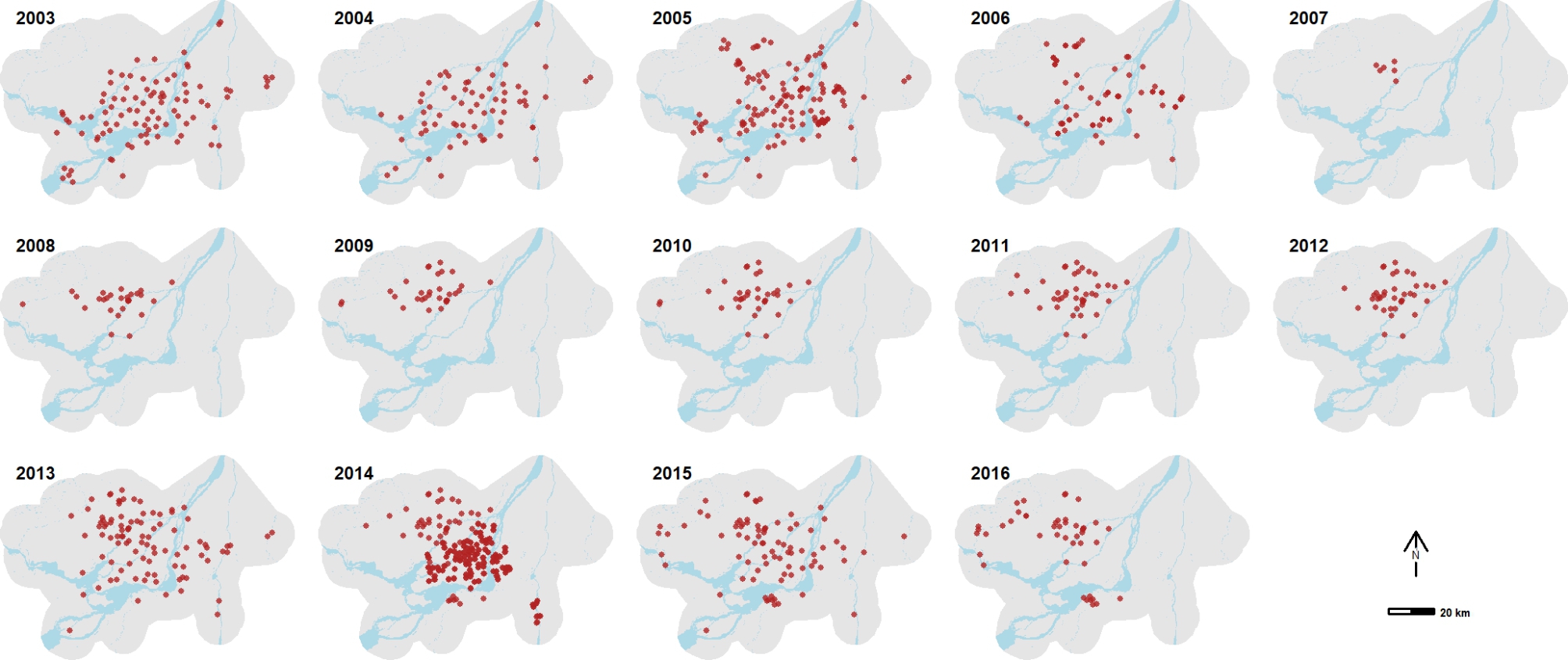
Location of mosquito traps deployed for each season across the Greater Montréal area, Quebec, Canada (2003–2016). All trap locations are shown even though some traps were not deployed for the whole mosquito season each year

**Fig. 3:**
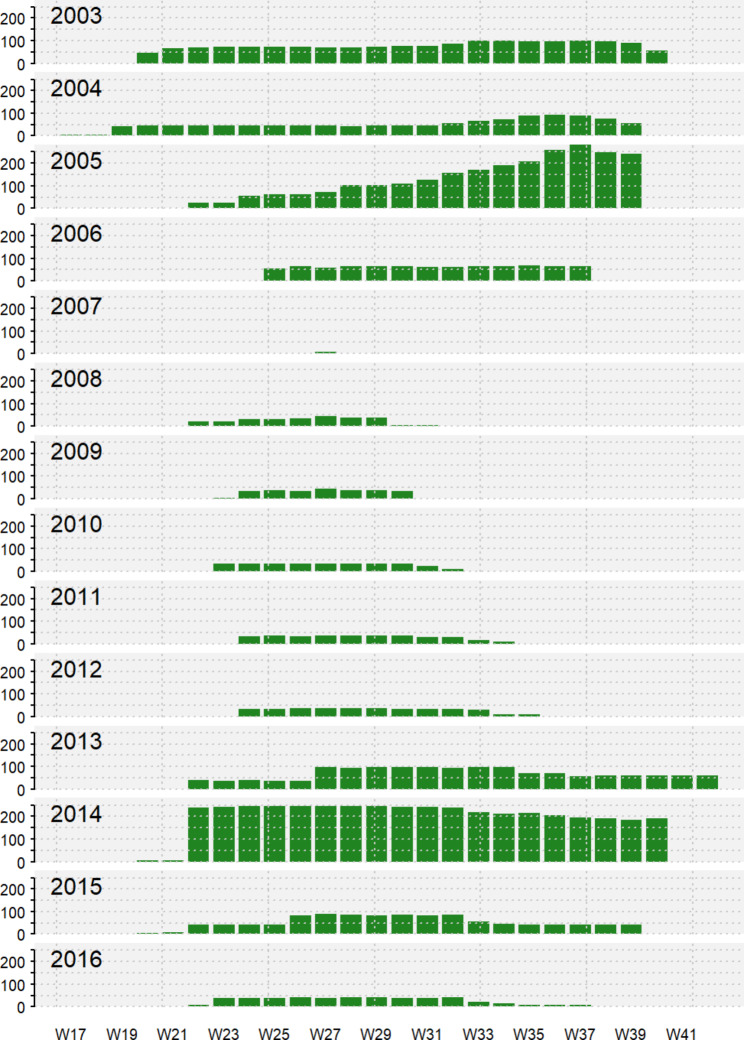
Number of mosquito vector traps deployed per year and week across the study period around the Greater Montréal area, Quebec, Canada (2003–2016). Traps deployed on different dates were grouped according to the week in which they were deployed

We selected four species or groups of species that pose a potential public health risk because of their respective vector competence for arboviruses: CPR, CQP, VEX and SMG. *Culex pipiens* and *Culex restuans* were lumped into a single group *Cx. pipiens-restuans* (CPR) due to their morphological similarity. *Oc. stimulans*, *Oc. fitchii* and *Oc.riparius* are also grouped together for this reason (SMG group). In this last group, because of the geographic distribution of the three species and their relative abundance, we can reasonably consider that the main or most abundant species in the SMG group is *Oc. stimulans* [42]. The mosquito catch data came partly from the provincial program under the Institut national de santé publique du Québec (INSPQ) and Quebec’s Ministère de la Santé et des Services sociaux (MSSS) for the surveillance of mosquito-borne diseases in Quebec, and partly from the Public Health Agency of Canada (PHAC) using the database of a private company involved in mosquito control (GDG Environnement Ltd.). Trapping was conducted with Center for Disease Control (CDC) CO_2_ light traps and mosquitoes were collected at each site at night. The dataset covers the years 2003 to 2016, but the number of traps deployed from year to year varied and fewer traps were installed in some years and in more geographically restricted areas (Fig. 2). The number of traps deployed also varied from week to week (Fig. 3). Trapped mosquitoes were identified and counted by species.

### Variables

Variables used for modelling mosquito abundances included weather and land-cover class variables (see below for a description). Seasonality in mosquito abundance patterns was taken into account by using day-of-year as an explanatory variable in all models as it integrates a lot of information relevant to mosquito development and abundance cycles (see the *Statistical Modelling* section below for details of how it was entered in the models).

#### Weather conditions

Weather variables were obtained from the Daymet estimates of daily surface weather data on a 1-km grid for North America [53] using the daymetr package [54]. Prior to extraction, we aggregated the 1 × 1 km cells to 2 × 2 km cells to reduce computation time and to fill in the few cells with missing data. Since only the daily maximum and minimum temperature are available from the Daymet data, we estimated the daily mean temperature by calculating the mean of the daily maximum and minimum temperatures. For each trap catch, we extracted the daily mean temperature and the daily precipitation (prcp). Because of the strong seasonal trend in the daily mean temperature and the high correlation between day-of-year and temperature, we removed the seasonal trend in the mean temperature to consider temperature anomalies instead (anom). Specifically, we calculated for each pixel the difference between the daily mean temperature and the mean daily mean temperature for the period from 2003 to 2016. We calculated the pluriannual daily mean temperature across the study period using a generalized additive model (GAM) with the daily mean temperature as our response variable and a smooth function with a cyclic basis on the day-of-year as our predictive variable. Temperature anomalies were obtained by subtracting the daily mean temperatures from the predicted daily mean temperatures. The R package mgcv [55] was used to build the GAM models. We did not consider precipitation anomalies as the seasonal trend in precipitations was much less pronounced. Finally, to take into account the effect of previous weather conditions on mosquito abundance, we considered lagged effects of daily temperature anomalies and precipitations by calculating the mean of the variables over the previous 2, 7, 30 and 90 days counting from the date of each catch. We thus had the current (two days) and the previous (7, 30 and 90 days) weather conditions as predictors of mosquito abundance. The weather conditions over two days correspond to the weather conditions on the day the trap is set and the day it is lifted.

#### Land cover

The first category of environmental data used is the land-cover class (LCC) layers derived from previously classified satellite (raster) data and vector data (Table 1**).** Eight LCCs were selected initially for our exercise based on our literature review: aquatic area (shallow and deep water), bare soil, urban/builtup, wetland, agricultural land, pasture/grassland, vegetated non-treed, and woodland. The raster layer processed by Agriculture and Agri-Food Canada (AAFC) [56] was used as a general base, and its classification was improved by the addition of multi-source data: the classified SPOT 4–5 raster land cover of Canada south of the treeline provided by the Government of Canada (GoC) [57] and two other layers in vector format were used for this purpose, namely the Ducks Unlimited Canada (DUC) [58] wetland map and Natural Resources Canada’s National Hydrographic Network (NHN) [59]. The different sources of LCC data were combined using the following decision rule: in non-urban areas, the information provided by the AAFC layer prevailed. In ‘urban’ areas, the information provided by the SPOT image prevailed over the AAFC image. The LCC was classified as a wetland if and only if the data provided by AAFC, SPOT and DUC all indicated the presence of a wetland. The LCC was classified as an aquatic area based on the NHN information, which prevailed over all other data sources.

**Table 1:** Sources and characteristics of land-cover and land-use variables for modelling the abundance and spatial distribution of Culex pipiens-restuans group, Aedes vexans, Coquilettidia perturbans and Ochlerotatus stimulans group in southern Quebec

Type of data	Source	Years acquired	Accuracy	Computed variables and units
Classified images	AAFC Annual Crop Inventory, GoC https://open.canada.ca/data/en/dataset/ba2645d5-4458-414d-b196-6303ac06c1c9	2011 (1 image)	30 m (overall classification accuracy of 85%)	Land-cover classes (%)
	Classified SPOT Land Cover of Canada South of Treeline, NRCAN, GoC https://open.canada.ca/data/en/dataset/d1fc6010-e2e7-401a-8dc1-544cd2ac0b03	Acquired between 2005 and 2010 (1 image)	20 m (overall classification accuracy of 71%)	
Vector data	DUC WL https://www.donneesquebec.ca/recherche/fr/dataset/milieux-humides-du-quebec	Identified between 1999 and 2005 (1 shapefile)	1:20,000	
	NHN, NRCAN, GoC https://www.nrcan.gc.ca/science-and-data/science-and-research/earth-sciences/geography/topographic-information/geobase-surface-water-program-geeau/national-hydrographic-network/21361	Identified between 1999 and 2014 (1 shapefile)	1:20,000	

AAFC: Agriculture and Agri-Food Canada; PHAC: Public Health Agency of Canada; DUC: Ducks Unlimited Canada; GoC: Government of Canada; WL: Wetlands; NHN: National Hydrographic Network

Although we had access to several land cover classes, trap locations were not necessarily chosen to cover the range of possible values for each class within the study area and most traps were installed near inhabited areas. Hence, we were not able to use some likely important classes for explaining mosquito abundance, such as wetland cover, which would have led to extrapolation beyond the range of observed values. We instead opted for using the three most abundant and variable classes in the study area, namely ‘urban’, ‘forest’ and ‘agriculture’ cover (**see** Fig. 1**)** for which we had good coverage and thus avoided extrapolation. We considered percentage of land cover around each trap at both a local (50 m buffer) and a regional (1 km buffer) scale. Although the environment immediately surrounding each trap likely has an important impact on the number of captures, the 30 m resolution of the land cover data prevented us from considering more local effects. Since the land-cover data used were not available for the years from 2003 to 2010, we used the land cover from the year 2011 and applied it to all traps from 2003 to 2016 on the assumption that changes in land cover were relatively small.

The analyses were performed in PCI Geomatica [60], ArcGIS [61].

### Statistical modelling

We modeled for each species/group the number of mosquitos captured for each trap night as a count using a negative binomial likelihood with a log link. We included a spatial component to account for unexplained variability in mosquito abundance across space and to take into account spatial autocorrelation. Spatial models were built using INLA [62] and the SPDE approach [63]. Briefly, INLA is an alternative to MCMC for estimating latent Gaussian models in a Bayesian framework. The SPDE approach facilitates the inclusion of spatial effects by approximating Gaussian random fields through a link with Gaussian Markov Random Field (GMRF). The method proved very useful for estimating large spatial and spatiotemporal models [64] and for disease mapping [65–67].

Penalized complexity (PC) priors were used to reduce complexity of the spatial component whenever the data did not warrant it [68, 69]. The PC priors used for the range parameter controlling the extent of the spatial dependence in mosquito counts represented a probability of 0.1 that the range is smaller than 5 km. The PC prior for the standard deviation of the spatial fields represented a probability of 0.1 that the standard deviation of the spatial fields is over 0.5. Since this standard deviation has to be exponentiated to go from the link to the response scale, it represents substantial variations in mosquito counts. Basically, unless warranted by the data, the PC priors used shrink the parameters toward a model where the spatial effect is small and operating at a large scale. The shape parameter of the Matérn covariance function was set to the default value of two. The maximum side length of the mesh triangles used to approximate the spatial component of the model in the SPDE approach was set to 0.5 km. The details of mesh construction can be found in the R code available upon request.

All our models had day-of-year and a mix of weather and land-cover variables as predictors (see below for details of the model selection process). Natural splines were used to model the effect of day-of-year and to account for non-linearities in mosquito abundances across the season (function *ns* in R package *splines*). To facilitate the process of modelling and generating predictions with INLA, we specified the location of knots used for the splines. We subjectively chose a number of knots (10) that seemed to show a good compromise between capturing the main pattern of variation across the season while avoiding unlikely complexities. We used relatively tight normal priors on spline coefficients to facilitate model convergence (mean = 0, sd = 1). Prior to model formulation, multicollinearity between predictors was assessed using pairwise correlations and variance inflation factors (VIF) with the car R package [70]. We avoided using the three land-cover classes as VIF values were over three when all classes were used in the same models. Thus, for each species, we chose the two land-cover classes most likely to have an influence on abundance according to the literature and to the biology of the species, naming ‘agriculture’ and ‘forest’ for VEX, and ‘urban’ and ‘forest’ for all the other species or group of species. Predictors included in a given model either had a correlation lower than 0.7 with any other predictor and had VIF values below three. Due to high correlation between mean temperature at longer time lags (i.e. 30, 90 days) and day-of-year, we avoided including both day-of-year and mean temperatures in models and instead used anomaly values which lacked a seasonal trend.

All variables were scaled to have a mean of 0 and a standard deviation of 1 prior to analysis. Wide normal priors were used for all variables (mean = 0, sd = 30). An offset was used to account for the number of nights a trap was deployed, although the vast majority of traps were deployed for a single night each time. Models were estimated with the R package INLA (v. 22.01.25) using the experimental mode [71] and in R 4.1.2 (R Core Team 2021). The following packages were use for spatial tasks and figure constructions: sp, sf, terra, exactextractr magick, rnaturalearth. The code is available on request.

#### Model selection

Model selection was used to determine at which spatial or temporal scale weather and land-cover variables operate. For each species, we built a series of models that tested whether local and/or regional land cover and current and/or previous weather were better predictors of mosquito abundance. We built 3 sets of landcover variables (‘urban50 + forest50’, ‘urban1000 + forest1000’ and ‘urban50 + forest50 + urban1000 + forest1000’) where 50 refers to local scale and 1000 to regional scale. We also built 7 sets of weather variables (‘anom2 + prcp2’, ‘anom7 + prcp7’, ‘anom30 + prcp30’, ‘anom90 + prcp90’, ‘anom2 + prcp2 + anom7 + prcp7’, ‘anom2 + prcp2 + anom30 + prcp30’,’ anom2 + prcp2 + anom90 + prcp90’) where 2, 7, 30 and 90 refers to the previous days counting from the date of each catch. The combination of these 2 sets of variables brings us to 3 × 7 = 21 models for each species.

We compared each model within a set using the deviance information criterion (DIC), choosing the best model whenever a model clearly stood out or the most complete model when several models had similar DIC scores. Although the Watanabe-Akaike information criterion (WAIC) is considered superior to the DIC as it uses the entire posterior distribution, it is not recommended for models where there is dependence between observations [72]. We therefore used the DIC, keeping in mind that it is one of many methods and that model selection remains an open question [73]. To evaluate the usefulness of the spatial component, we also included in the set of models a model with the same explanatory variables as in the best model, but with the spatial component removed. To determine whether the importance of the spatial component was partly driven by the many explanatory variables we could not consider within a single model, we also considered a model with all explanatory variables, again with the spatial component removed.

#### Model checking and validation

Model fit was assessed through simulations by comparing data simulated from the models with the observed data. Histograms of simulated and observed count values were visually compared and showed an acceptable fit. The DHARMa package [74] was also used to assess zero-inflation and model fit through randomized quantile residuals [75]. Only the best model for each species was used to assess model fit. For all models, observed values were comparable and did not differ substantially from simulated values. Although many trap counts were zero, there was no indication of substantial zero-inflation with our data and preliminary analyses showed zero-inflation probabilities very close to zero. It is our interpretation that the negative binomial distribution used was able to cope with the number of zeros along with the smooth function used on day-of-year and the spatial component. We used the correlation between observed and predicted counts as a pseudo-R square measure and as a rough estimate of the explanatory power of the models. We compared the best spatial model with its non-spatial version to estimate the importance of the spatial component in explaining variations in abundance. We also compared both models with the non-spatial model containing all explanatory variables to determine whether the spatial component importance was mainly due to missing explanatory variables that we had considered for this study.

## Results

**Fig. 4:**
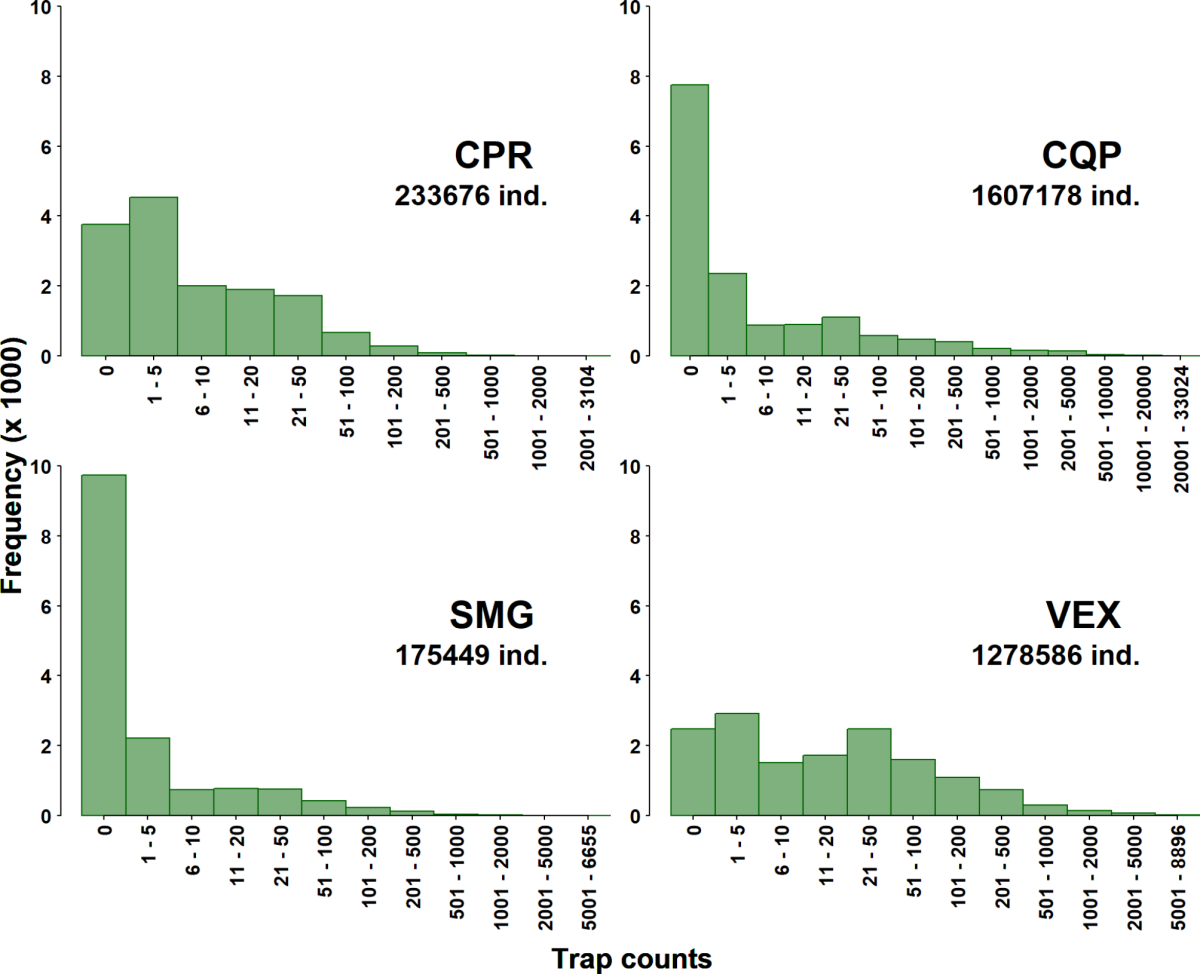
Mosquito count frequencies from traps deployed in the Greater Montréal area, Quebec, Canada (2003–2016). The total number of individuals captured is shown below the species code and the maximum number captured in a single trap night is shown on the X axis. The species/groups are CPR (Culex pipiens-restuans group), CQP (Coquillettidia_perturbans), SMG (Ochlerotatus stimulans-fitchii-riparius group) and VEX (Aedes vexans)

More than 3 million mosquitos were captured in the ~ 14,000 trap nights (Fig. 4). The maximum count for a single trap night for each species/group varied from 3,104 (CPR) to 33,024 (CQP).

**Fig. 5:**
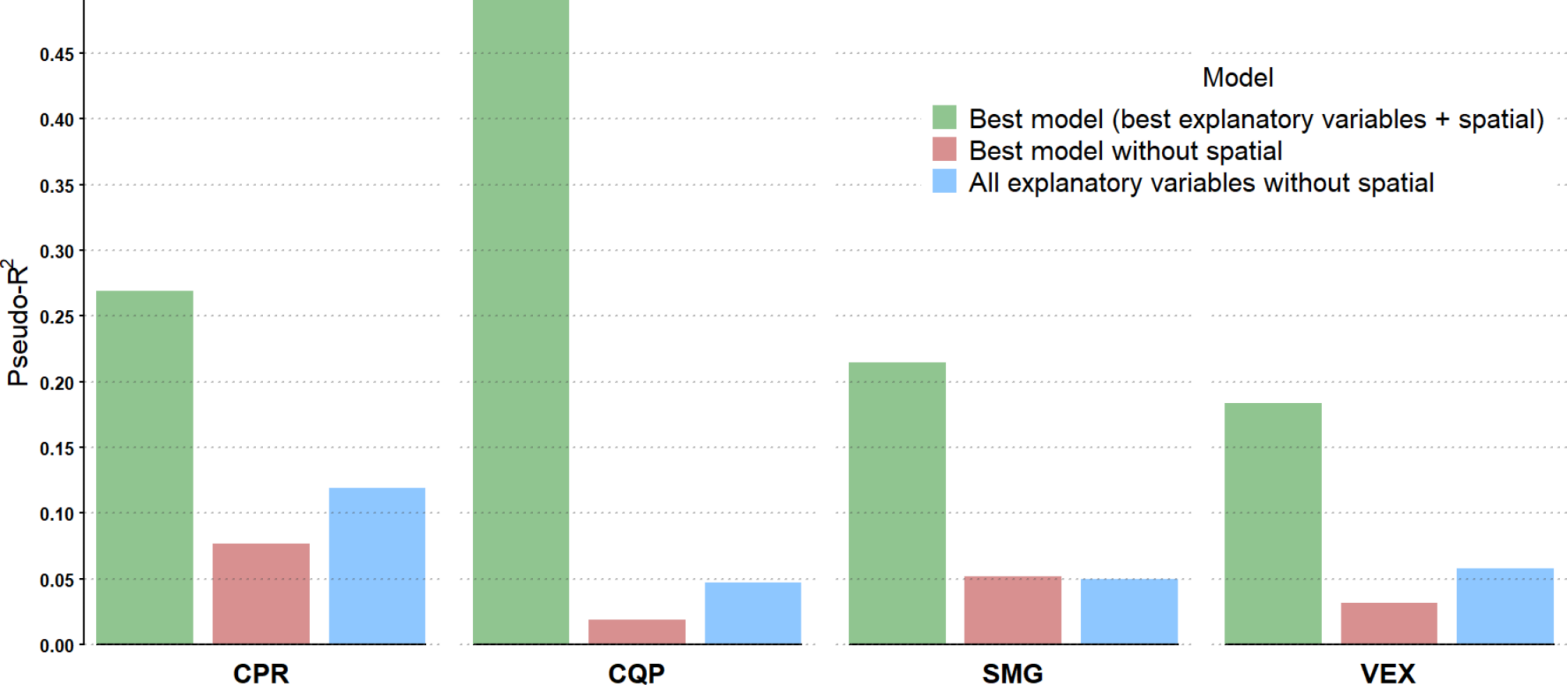
Pseudo-R square of models used for modelling mosquito counts in traps deployed in the Greater Montréal area, Quebec, Canada (2013 − 206). Models were estimated with a Bayesian negative binomial spatial model with the number of mosquitos trapped as a response variable. For each species, the R square of the best model is shown in green. For comparison, the R square of models with the spatial component removed are also shown; the best model with only explanatory variables is shown in red, and a model with all potential explanatory variables is shown in blue. The species/groups are CPR (*Culex pipiens-restuans* group), CQP (*Coquillettidia perturbans*), SMG (*Ochlerotatus stimulans-fitchii-riparius* group) and VEX (*Aedes vexans*)

For most species, models containing both local and regional land-cover variables and both current and previous weather variables were selected as the best model, except for CQP, where the best model only contained the regional land-cover variables (Table 2). The weather conditions during the trapping day and previous weather conditions summarized over 30 or 90 days were preferred over a shorter period of seven days, suggesting current and long-term previous weather conditions effects on mosquito abundance. In all cases, the best model with the spatial component removed performed worse than any other model including the spatial component. This is also reflected in the pseudo-R square, where the best spatial model always had a pseudo-R square at least three times higher than the corresponding non-spatial model (Fig. 5). Models with the spatial component have interesting pseudo-R square values, especially for CQP, which is having a pseudo-R square of more than 45%. In general, non-spatial models with the best explanatory variables had relatively low pseudo-R square values, suggesting that variations in mosquito abundance were marginally explained by our explanatory variables. Except for CQP, non-spatial models with all explanatory variables had pseudo-R square values much lower than the best spatial model, suggesting that the importance of the spatial component was not entirely due to the limited amount of variables we could consider in each model.

**Table 2:** Model section and relative DIC (deviance information criterion) values for each mosquito species/group modeled. Mosquito counts in each trap deployed in the Greater Montréal area, Quebec, Canada (2003–2016) were modelled using a Bayesian negative binomial spatial model. Day-of-year was modelled using a natural spline. The species/groups are CPR (*Culex pipiens-restuans* group), CQP (*Coquillettidia_perturbans*), SMG (*Ochlerotatus stimulans-fitchii-riparius* group) and VEX (*Aedes vexans*). ‘ns’ refers to ‘nonspatial’

CPR		
*Model*	*Variables*	*ΔDIC*
CPR16	ns(doy) + anom2 + anom30 + prcp2 + prcp30 + forest50 + urban50 + spatial	0.00
CPR18	ns(doy) + anom2 + anom30 + prcp2 + prcp30 + forest50 + forest1000 + urban50 + urban1000 + spatial	10.72
CPR17	ns(doy) + anom2 + anom30 + prcp2 + prcp30 + forest1000 + urban1000 + spatial	13.88
CPR07	ns(doy) + anom30 + prcp30 + forest50 + urban50 + spatial	43.80
CPR09	ns(doy) + anom30 + prcp30 + forest50 + forest1000 + urban50 + urban1000 + spatial	50.48
CPR08	ns(doy) + anom30 + prcp30 + forest1000 + urban1000 + spatial	50.61
CPR21	ns(doy) + anom2 + anom90 + prcp2 + prcp90 + forest50 + forest1000 + urban50 + urban1000 + spatial	56.90
CPR19	ns(doy) + anom2 + anom90 + prcp2 + prcp90 + forest50 + urban50 + spatial	57.62
CPR20	ns(doy) + anom2 + anom90 + prcp2 + prcp90 + forest1000 + urban1000 + spatial	65.51
CPR15	ns(doy) + anom2 + anom7 + prcp2 + prcp7 + forest50 + forest1000 + urban50 + urban1000 + spatial	72.47
CPR13	ns(doy) + anom2 + anom7 + prcp2 + prcp7 + forest50 + urban50 + spatial	76.63
CPR01	ns(doy) + anom2 + prcp2 + forest50 + urban50 + spatial	82.75
CPR14	ns(doy) + anom2 + anom7 + prcp2 + prcp7 + forest1000 + urban1000 + spatial	82.91
CPR06	ns(doy) + anom7 + prcp7 + forest50 + forest1000 + urban50 + urban1000 + spatial	87.24
CPR03	ns(doy) + anom2 + prcp2 + forest50 + forest1000 + urban50 + urban1000 + spatial	87.61
CPR02	ns(doy) + anom2 + prcp2 + forest1000 + urban1000 + spatial	89.14
CPR04	ns(doy) + anom7 + prcp7 + forest50 + urban50 + spatial	90.46
CPR05	ns(doy) + anom7 + prcp7 + forest1000 + urban1000 + spatial	96.02
CPR12	ns(doy) + anom90 + prcp90 + forest50 + forest1000 + urban50 + urban1000 + spatial	128.00
CPR11	ns(doy) + anom90 + prcp90 + forest1000 + urban1000 + spatial	133.37
CPR10	ns(doy) + anom90 + prcp90 + forest50 + urban50 + spatial	137.08
CPR16ns	ns(doy) + anom2 + anom30 + prcp2 + prcp30 + forest50 + urban50	3524.14
**CQP**		
***Model***	***Variables***	***ΔDIC***
CQP20	ns(doy) + anom2 + anom90 + prcp2 + prcp90 + forest1000 + urban1000 + spatial	0.00
CQP21	ns(doy) + anom2 + anom90 + prcp2 + prcp90 + forest50 + forest1000 + urban50 + urban1000 + spatial	8.19
CQP19	ns(doy) + anom2 + anom90 + prcp2 + prcp90 + forest50 + urban50 + spatial	13.81
CQP17	ns(doy) + anom2 + anom30 + prcp2 + prcp30 + forest1000 + urban1000 + spatial	63.40
CQP11	ns(doy) + anom90 + prcp90 + forest1000 + urban1000 + spatial	66.82
CQP18	ns(doy) + anom2 + anom30 + prcp2 + prcp30 + forest50 + forest1000 + urban50 + urban1000 + spatial	73.73
CQP12	ns(doy) + anom90 + prcp90 + forest50 + forest1000 + urban50 + urban1000 + spatial	76.00
CQP16	ns(doy) + anom2 + anom30 + prcp2 + prcp30 + forest50 + urban50	79.78
CQP10	ns(doy) + anom90 + prcp90 + forest50 + urban50 + spatial	81.80
CQP14	ns(doy) + anom2 + anom7 + prcp2 + prcp7 + forest1000 + urban1000 + spatial	94.89
CQP02	ns(doy) + anom2 + prcp2 + forest1000 + urban1000 + spatial	96.94
CQP15	ns(doy) + anom2 + anom7 + prcp2 + prcp7 + forest50 + forest1000 + urban50 + urban1000 + spatial	104.93
CQP03	ns(doy) + anom2 + prcp2 + forest50 + forest1000 + urban50 + urban1000 + spatial	106.98
CQP13	ns(doy) + anom2 + anom7 + prcp2 + prcp7 + forest50 + urban50 + spatial	111.09
CQP01	ns(doy) + anom2 + prcp2 + forest50 + urban50 + spatial	113.05
CQP08	ns(doy) + anom30 + prcp30 + forest1000 + urban1000 + spatial	148.90
CQP05	ns(doy) + anom7 + prcp7 + forest1000 + urban1000 + spatial	160.00
CQP09	ns(doy) + anom30 + prcp30 + forest50 + forest1000 + urban50 + urban1000 + spatial	160.01
CQP07	ns(doy) + anom30 + prcp30 + forest50 + urban50 + spatial	167.28
CQP06	ns(doy) + anom7 + prcp7 + forest50 + forest1000 + urban50 + urban1000 + spatial	169.89
CQP04	ns(doy) + anom7 + prcp7 + forest50 + urban50 + spatial	176.59
CQP20ns	ns(doy) + anom2 + anom90 + prcp2 + prcp90 + forest1000 + urban1000	8819.00
**SMG**		
***Model***	***Variables***	***ΔDIC***
SMG21	ns(doy) + anom2 + anom90 + prcp2 + prcp90 + forest50 + forest1000 + urban50 + urban1000 + spatial	0.00
SMG12	ns(doy) + anom90 + prcp90 + forest50 + forest1000 + urban50 + urban1000 + spatial	8.67
SMG19	ns(doy) + anom2 + anom90 + prcp2 + prcp90 + forest50 + urban50 + spatial	9.14
SMG10	ns(doy) + anom90 + prcp90 + forest50 + urban50 + spatial	16.54
SMG20	ns(doy) + anom2 + anom90 + prcp2 + prcp90 + forest1000 + urban1000 + spatial	21.50
SMG11	ns(doy) + anom90 + prcp90 + forest1000 + urban1000 + spatial	28.57
SMG03	ns(doy) + anom2 + prcp2 + forest50 + forest1000 + urban50 + urban1000 + spatial	92.58
SMG18	ns(doy) + anom2 + anom30 + prcp2 + prcp30 + forest50 + forest1000 + urban50 + urban1000 + spatial	93.52
SMG15	ns(doy) + anom2 + anom7 + prcp2 + prcp7 + forest50 + forest1000 + urban50 + urban1000 + spatial	94.69
SMG09	ns(doy) + anom30 + prcp30 + forest50 + forest1000 + urban50 + urban1000 + spatial	96.39
SMG16	ns(doy) + anom2 + anom30 + prcp2 + prcp30 + forest50 + urban50	100.83
SMG06	ns(doy) + anom7 + prcp7 + forest50 + forest1000 + urban50 + urban1000 + spatial	103.49
SMG07	ns(doy) + anom30 + prcp30 + forest50 + urban50 + spatial	105.51
SMG01	ns(doy) + anom2 + prcp2 + forest50 + urban50 + spatial	108.46
SMG02	ns(doy) + anom2 + prcp2 + forest1000 + urban1000 + spatial	109.58
SMG13	ns(doy) + anom2 + anom7 + prcp2 + prcp7 + forest50 + urban50 + spatial	109.94
SMG04	ns(doy) + anom7 + prcp7 + forest50 + urban50 + spatial	110.30
SMG14	ns(doy) + anom2 + anom7 + prcp2 + prcp7 + forest1000 + urban1000 + spatial	111.94
SMG17	ns(doy) + anom2 + anom30 + prcp2 + prcp30 + forest1000 + urban1000 + spatial	112.07
SMG08	ns(doy) + anom30 + prcp30 + forest1000 + urban1000 + spatial	116.78
SMG05	ns(doy) + anom7 + prcp7 + forest1000 + urban1000 + spatial	122.62
SMG21ns	ns(doy) + anom2 + anom90 + prcp2 + prcp90 + forest50 + forest1000 + urban50 + urban1000	4450.21
**VEX**		
***Model***	***Variables***	***ΔDIC***
VEX18	ns(doy) + anom2 + anom30 + prcp2 + prcp30 + agriculture50 + agriculture1000 + forest50 + forest1000 + spatial	0.00
VEX17	ns(doy) + anom2 + anom30 + prcp2 + prcp30 + forest1000 + agriculture1000 + spatial	3.64
VEX16	ns(doy) + anom2 + anom30 + prcp2 + prcp30 + forest50 + agriculture50	6.23
VEX08	ns(doy) + anom30 + prcp30 + forest1000 + agriculture1000 + spatial	645.03
VEX09	ns(doy) + anom30 + prcp30 + forest50 + forest1000 + agriculture50 + agriculture1000 + spatial	645.76
VEX07	ns(doy) + anom30 + prcp30 + forest50 + agriculture50 + spatial	652.08
VEX20	ns(doy) + anom2 + anom90 + prcp2 + prcp90 + forest1000 + agriculture1000 + spatial	1019.44
VEX19	ns(doy) + anom2 + anom90 + prcp2 + prcp90 + forest50 + agriculture50 + spatial	1029.01
VEX21	ns(doy) + anom2 + anom90 + prcp2 + prcp90 + forest50 + forest1000 + agriculture50 + agriculture1000 + spatial	1030.52
VEX13	ns(doy) + anom2 + anom7 + prcp2 + prcp7 + forest50 + agriculture50 + spatial	1300.48
VEX15	ns(doy) + anom2 + anom7 + prcp2 + prcp7 + forest50 + forest1000 + agriculture50 + agriculture1000 + spatial	1300.85
VEX14	ns(doy) + anom2 + anom7 + prcp2 + prcp7 + forest1000 + agriculture1000 + spatial	1310.14
VEX02	ns(doy) + anom2 + prcp2 + forest1000 + agriculture1000 + spatial	1310.33
VEX03	ns(doy) + anom2 + prcp2 + forest50 + forest1000 + agriculture50 + agriculture1000 + spatial	1327.29
VEX01	ns(doy) + anom2 + prcp2 + forest50 + agriculture50 + spatial	1331.53
VEX10	ns(doy) + anom90 + prcp90 + forest50 + agriculture50 + spatial	1597.35
VEX12	ns(doy) + anom90 + prcp90 + forest50 + forest1000 + agriculture50 + agriculture1000 + spatial	1606.64
VEX11	ns(doy) + anom90 + prcp90 + forest1000 + agriculture1000 + spatial	1608.60
VEX05	ns(doy) + anom7 + prcp7 + forest1000 + agriculture1000 + spatial	1685.95
VEX06	ns(doy) + anom7 + prcp7 + forest50 + forest1000 + agriculture50 + agriculture1000 + spatial	1690.23
VEX04	ns(doy) + anom7 + prcp7 + forest50 + agriculture50 + spatial	1714.38
VEX18ns	ns(doy) + anom2 + anom30 + prcp2 + prcp30 + forest50 + forest1000 + agriculture50 + agriculture1000	4725.02

**Fig. 6:**
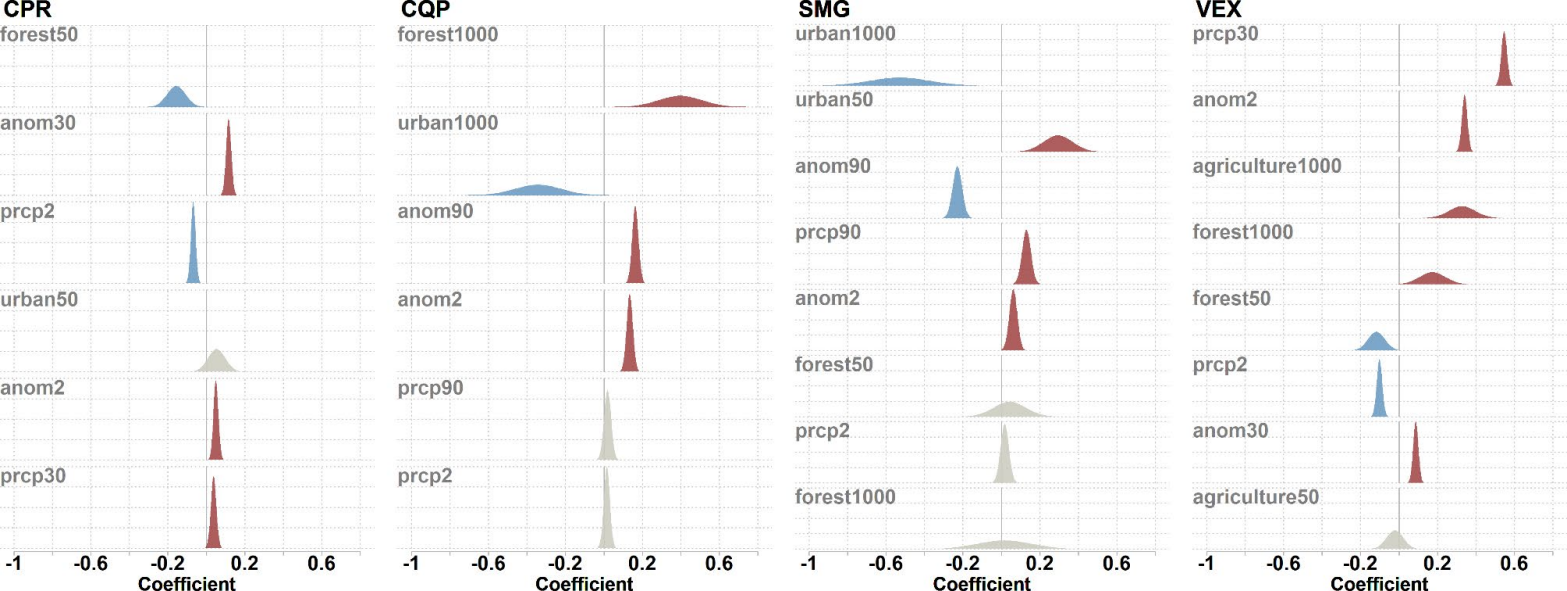
Posterior distributions of land-cover and weather coefficients used for modelling mosquito counts in traps deployed in the Greater Montréal area, Quebec, Canada (2013 − 206). Coefficients were estimated using a Bayesian negative binomial spatial model with the number of mosquitos trapped as a response variable. For each species, coefficients are from the best model selected by DIC. Explanatory variables were scaled prior to analysis, making coefficients comparable. Variables are ranked in decreasing order according to the mode of each posterior distribution (in absolute value). Negative coefficients are shown in blue, positive coefficients are shown in red and coefficients for which the 95% CI contains zero are shown in gray. The species/groups are CPR (*Culex pipiens-restuans* group), CQP (*Coquillettidia perturbans*), SMG (*Ochlerotatus stimulans-fitchii-riparius* group) and VEX (*Aedes vexans*)

Some of the weather variables retained in the best model were averaged for either the 30 (CPR, VEX) or 90 (CQP, SMG) previous days, indicating that previous weather can have a cumulative effect on mosquito abundance (Table 2). However, most effects were relatively weak, except for VEX, where higher precipitations had a strong positive effect on counts (Fig. 6). Current weather also seemed to have some effects on abundance, especially for VEX abundance, which is positively associated with high temperature during the day of capture. High precipitation on the day of capture seems to have a weak effect on diminishing the abundance for CPR and VEX. Precipitation over 90 days before capture has a strong positive effect on SMG abundance.

In general, land-cover variables had a relatively weak effect on abundance. In addition, land-cover variables measured at a regional scale (1 km) had a stronger effect on abundance when compared with the land cover measured on a local scale (50 m). For CPR, abundance decreases slightly with the presence of forest at the local scale. For CQP, abundance strongly increased with the percentage of forests and strongly decreased with the percentage of ‘urban’ land cover within 1 km. SMG abundances decreased with ‘urban’ land cover and the regional scale but increase with the same landcover class, ‘urban’, at the local scale. Finally, VEX abundances increased with ‘forest’ and ‘agriculture’ land cover at the regional scale.

**Fig. 7:**
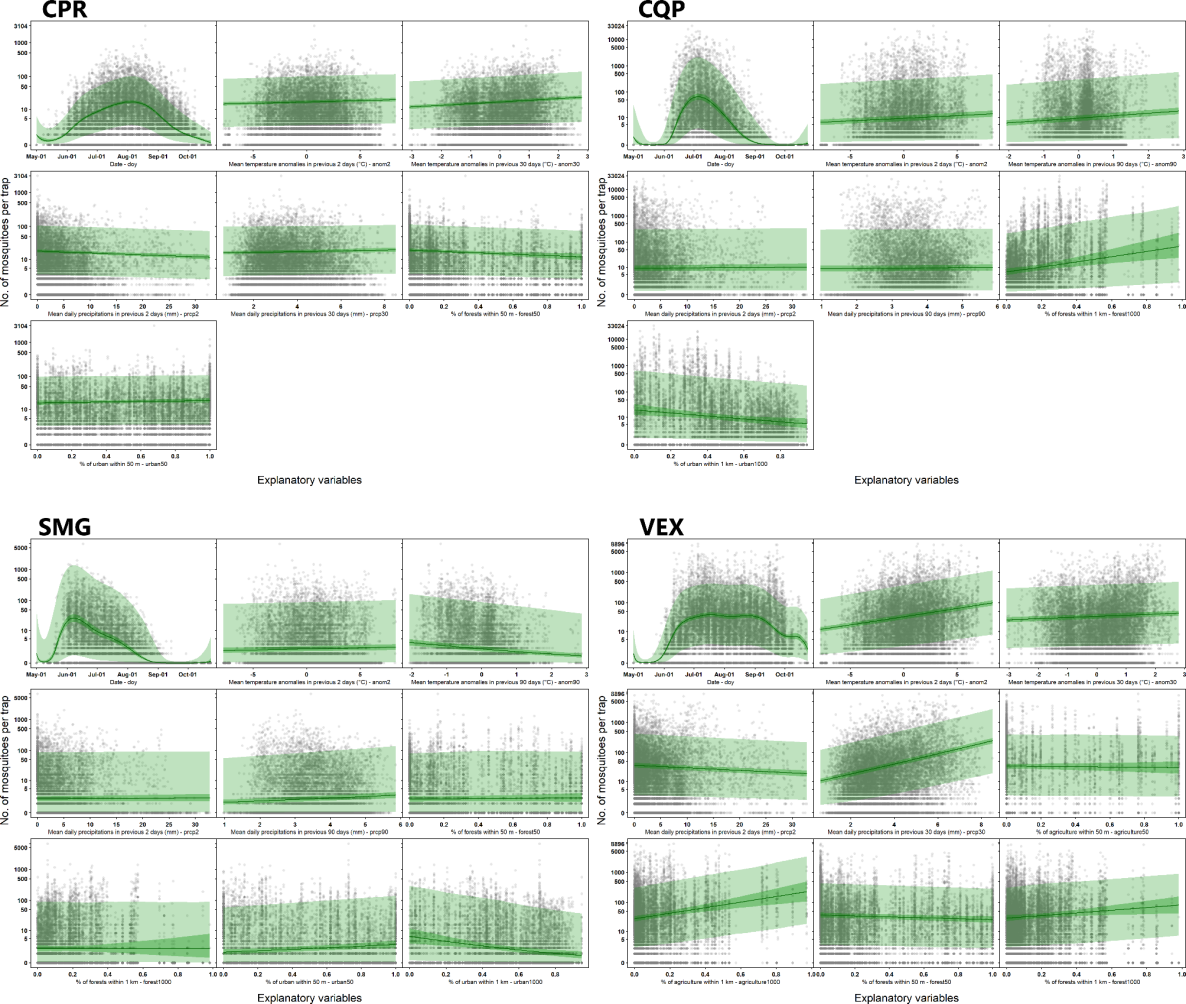
Effects of explanatory variables on mosquito counts in traps deployed in the Greater Montréal area, Quebec, Canada (2013 − 206). Predictions are derived from a Bayesian negative binomial spatial model with the number of mosquitos trapped. For each species, the best model was used to generate predictions. The darker 95% CIs (credible intervals) show the posterior distributions of predictions when only taking into account the fixed component of the model. The larger 95% CI shows the posterior distribution of predictions when the spatial part of the model is also included. The location used for generating the spatial component is far from the observed data and is characterized by a high uncertainty. Variables not displayed on a given graph were fixed to their mean values when generating predictions. Mosquito counts are shown on a log scale to facilitate visualization. To add counts of 0 to the figure, a value of one was added to the observed counts, but the Y axis labels display the observed counts. The species/groups are CPR (*Culex pipiens-restuans* group), CQP (*Coquillettidia perturbans*), SMG (*Ochlerotatus stimulans-fitchii-riparius* group) and VEX (*Aedes vexans*)

For all species, seasonality (day-of-year) seemed to have the largest effect on abundance. For CQP and SMG, the peak in abundance appears more concentrated compared with CPR and VEX, for which the peak in abundance appears more spread out across the season (Fig. 7). Contrary to other species, SMG abundances are characterized by an earlier and shorter season compared with other species.

**Fig. 8:**
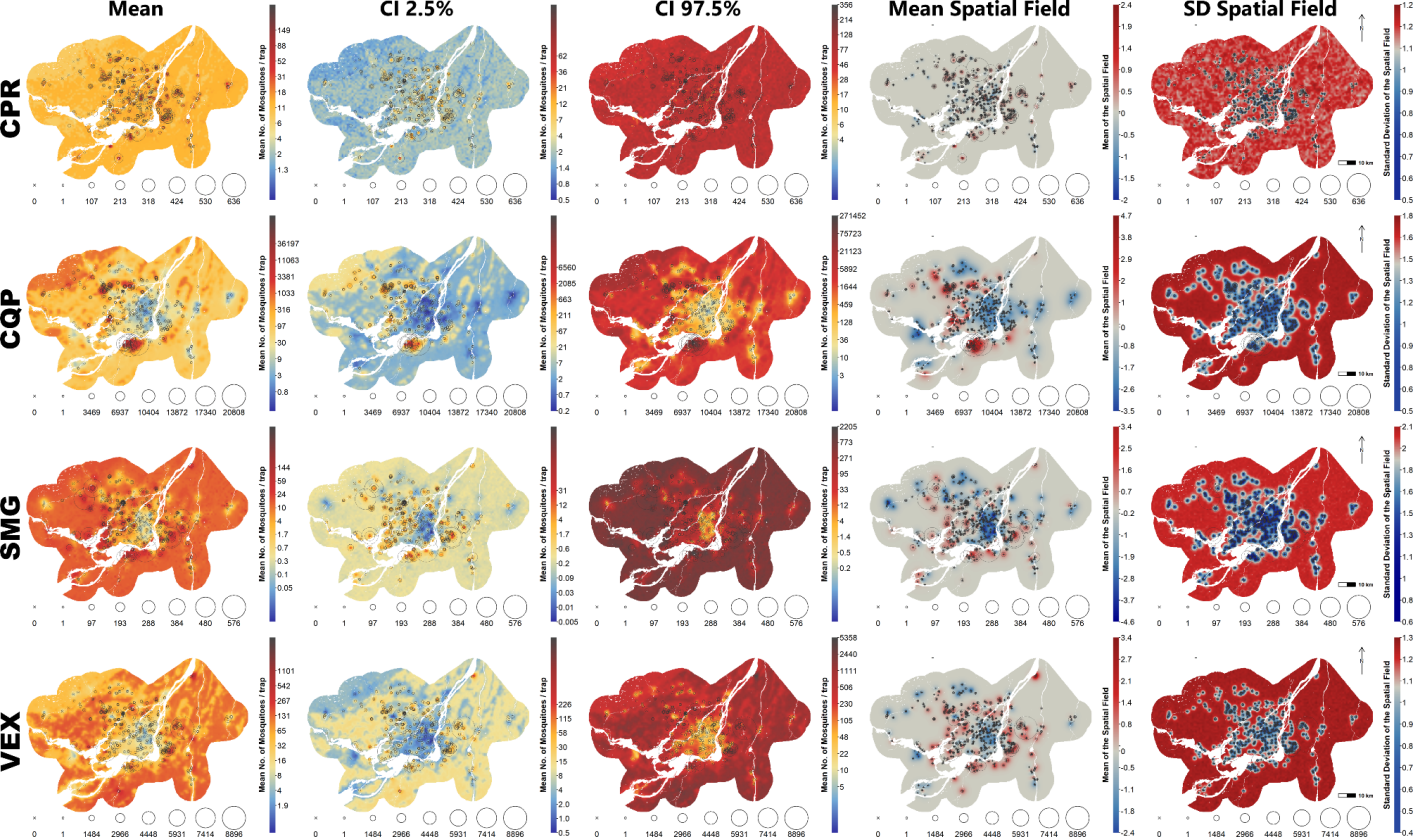
Predicted mean, 2.5–97.5% CI (credible interval) and spatial component of modelled counts of mosquitos across the Greater Montréal area, Quebec, Canada (2003–2016). Counts were modelled with a Bayesian negative binomial spatial model with date, weather and land-cover characteristics as explanatory variables. The first three columns (Mean, CI 2.5% and CI 97.5%) show the mean and lower and upper bounds of the 95% CI of predicted counts. The colours scale is the same for the three maps and its range is determined by the lowest predicted value and the highest predicted or observed value around the chosen date (July 10). The tick marks on the scale show the minimum and the maximum counts predicted for a given map. All observations shown are within three days of July 10 and are drawn with a size proportional to the counts observed. Counts of 0 are shown with an “x.” Predictions were generated by keeping the weather variables fixed to their mean values for each pixel. Variations in predicted counts are thus entirely due to landscape variables and to the spatial component. The last two columns show the spatial field and its standard deviation on the link scale. Positive values of the spatial field indicate areas with higher-than-expected counts and negative values indicate areas with lower-than-expected counts. In addition to the observations shown in the previous three columns, all trap locations are shown with an “x” to emphasis their influence on the spatial field. The species/groups are CPR (*Culex pipiens-restuans* group), CQP (*Coquillettidia perturbans*), SMG (*Ochlerotatus stimulans-fitchii-riparius* group) and VEX (*Aedes vexans*)

**Fig. 9:**
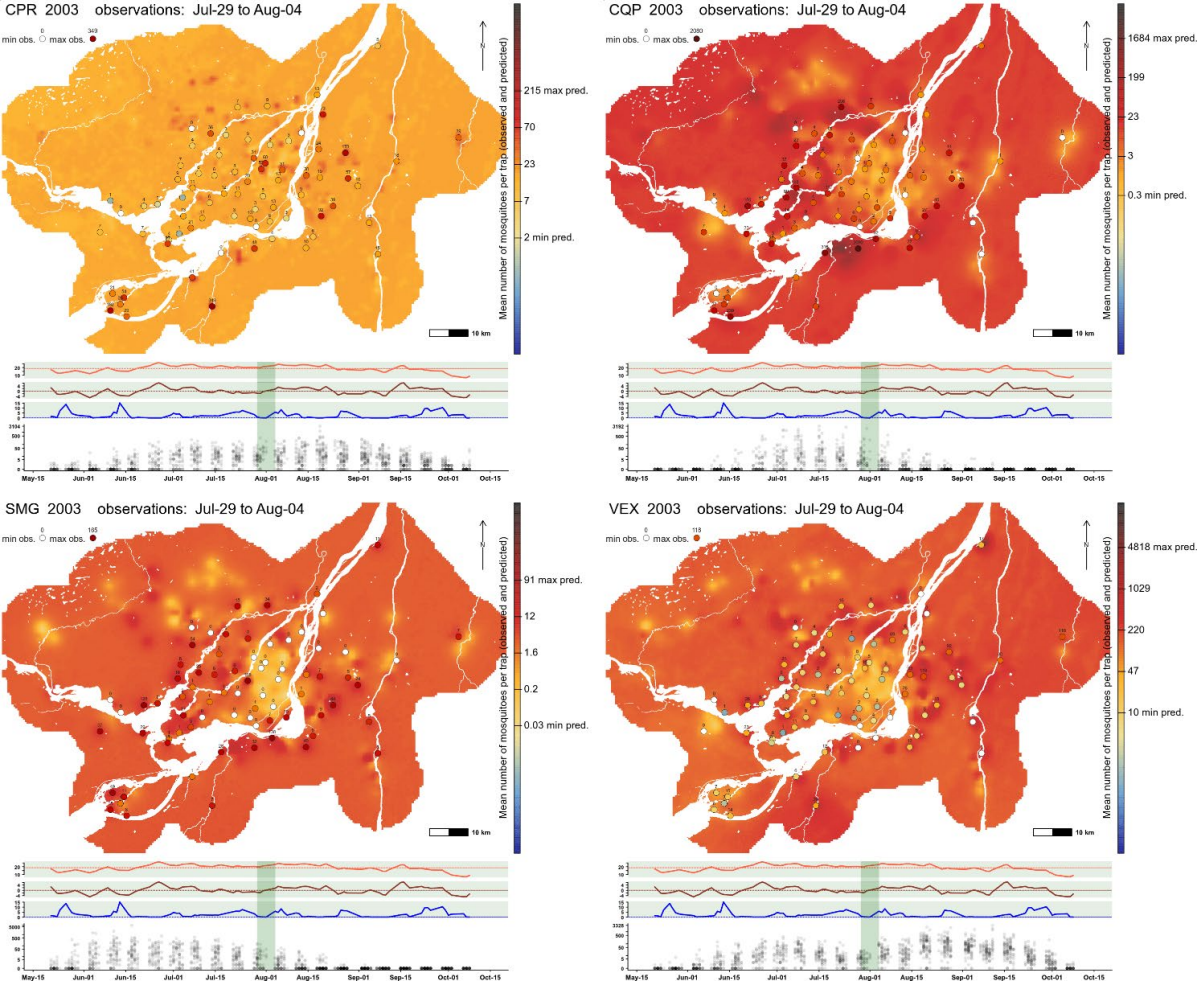
Predicted mosquito counts for the week July 29th – Aug4th 2003 season in the Greater Montréal area, Quebec, Canada. Predicted counts are derived from a Bayesian negative binomial spatial model with date, weather and land-cover characteristics as explanatory variables. The range of the colour scale is determined by the lowest count predicted and by the highest observed or predicted count. The tick marks show the minimum and maximum predicted counts for each map. Observations shown are within three days of the date used for a given map. Counts of zero are shown using the colour white. Current weather variables were used when generating predictions. Since land-cover variables were constant, variations in predicted counts are entirely due to the weather variables and the spatial component. The following three panels show variations in mean temperature, anomaly and precipitation over the previous two days across the whole study region (mean of tmean2, anom2 and prcp2). The dashed lines show the mean value for the temperature and the anomaly and the 0 mm for the precipitations. Finally, the bottom panel shows the observed counts on a log scale across the whole 2003 season. The species/groups are CPR (*Culex pipiens-restuans* group), CQP (*Coquillettidia perturbans*), SMG (*Ochlerotatus stimulans-fitchii-riparius* group) and VEX (*Aedes vexans*)

All mapped predictions were characterized by very wide credible intervals, which translates to very high uncertainties in predictions, especially when looking at predictions far from where the traps were installed (Fig. 8). Indeed, the standard deviation is high and more or less constant across a large part of the study area. Coupled with the relatively small range of the spatial field, predictions outside of areas where traps were deployed are characterized by large uncertainties. This is also easily seen in Fig. 7, where the spatial component accounts for the larger part of the uncertainty around predictions and, in most cases, the uncertainty associated with the spatial component appears more important than the variation associated with the explanatory variables. Nevertheless, maps show that there are very large variations in abundance across the study area associated with land-cover variables and the spatial component. For all species, abundances appear much lower than expected in the eastern and central part of Montréal, while abundances are higher in various areas in the western part and around the Island of Montréal. Figure 9 and Additional files 1, 2, 3 and 4 shows both the intra-seasonal and spatial variations, for the year 2003, of mosquito abundance, and it illustrates how the abundance of mosquitoes can be variable during the season and in space, depending on the landscape.

## Discussion

This paper led to the creation of maps of mean daily abundance for four mosquito species or species groups, namely CPR (*Culex pipiens-restuans* group), CQP (*Coquillettidia perturbans*), SMG (*Ochlerotatus stimulans-fitchii-riparius* group) and VEX (*Aedes vexans*), that are key to public health in southern Quebec. This type of map is of immediate interest to support decision-making for the management, control and prevention of mosquito-borne diseases. However, our modelling work also highlights challenges in modelling and mapping mosquito abundances. Despite incorporating environmental predictors at several spatial and temporal scales, the results obtained show that the variance explained by these variables remains low and that the unexplained variance remains predominant, as indicated by the strength of the spatial component and the high uncertainty in predicted abundance across the study area.

Globally, in the best models, we show the need for both current (for weather) or local (for land-cover), which describe the conditions of the day of capture and at the capture site, and the variables with a regional effect (for the land-cover variables) or cumulative effect (for weather variables), which describe what happened in the general habitat surrounding the trap or during the weeks prior to capture. Considering environmental variables measured at different temporal and spatial scales is already well established, although not always applied, and is a key aspect of mosquito abundance modelling [76]. We can easily explain the role of these different scales, in particular the temperature conditions or the precipitation over several days before the capture, which is likely linked to availabilities of breeding sites and to the speed of development of the pre-adult stages, and the temperature and precipitation during the day of capture, which influence the daily activity of adult mosquitoes.

VEX is the mosquito species for which the environmental predictors appeared most important. They include the positive role of high precipitation over the month preceding capture as well as the temperature on the day of capture. This is coherent with what is described in other studies realized in Quebec [22] and Ontario [36]. Furthermore, for VEX, increased abundance in agricultural landscapes is also well documented [34, 43]. The positive effect of ‘forest’ is less frequently found in the literature, although Hopkins et al. described VEX’s presence in forest areas subject to human disturbance [77], which corresponds to the type of forests we have in our study region.

The most important predictors for CPR had a relatively weak effect but underline the positive effect of higher-than-normal temperature during a period of one month prior to the day of capture, as well as the negative effect of precipitation on the day of capture. This is consistent with previous work realized in Quebec and Ontario [12, 22, 23, 36]. Negative impacts of ‘forest’ cover at the local scale can be compared with results observed in other studies that showed how an urbanized landscape increases the abundances of this species [17, 32, 33]. Indeed ‘forest’ could be considered as opposite of ‘urban’ in term of landscape. But again, this shows the difficulties of correctly describing these types of relationships with various spatial scales and data sources. We will come back to this point later.

For CQP, the environmental predictors with the largest effect are ‘forest’ land cover, at the regional scale, which tends to increase the mosquito abundance, and ‘urban’ land cover, again at the regional scale, which diminishes its abundance. These observations are hard to directly reconcile with the literature, as CQP is described as a mosquito species dependent on wetland, mainly because its larva requires permanent bogs and marshes to complete its life cycle [42, 45]. Unfortunately, wetland was not a land cover we were able to include in our analyses, because of the trapping design, as explained in the methods section. However, assuming that urban is negatively correlated to wetland, our results appear very coherent with the mosquito biology.

Finally, the most important predictors for the SMG group are the ‘urban’ land cover, at the regional and local scale, which respectively decreases and increases abundance, and the temperature anomalies over 90 days before capture, which negatively impact abundance. The SMG group consists of three species that are designated as “spring” species, abundant early in the season, just after the snow melts, and using forested vernal pools for breeding [17, 42, 49]. These biological characteristics are partially coherent with our best model if we hypothesize that forested vernal pools are less frequent in urban areas, at the regional scale. The positive correlation with ‘urban’ land cover at the local scale is more difficult to explain, although it could be associated with the existence in the mosquito environment of artificial breeding habitat that the mosquito is able to use.

Despite the coherence of our results with mosquito biology and other studies, the larger part of the variance was unexplained by our variables. This suggests that the weather and land-cover variables we used were not sufficient and we may have missed the most important variables for explaining variations in mosquito abundance, or simply have not characterized these variables at the right spatial or temporal scale.

By restricting the number of models and variables used, we may have failed to identify the most important combination of timescale at which weather conditions operate and the scale at which land-cover variables are the most important for mosquito habitat. Although we considered scales in this study, it was not our main objective and we were limited by our model selection approach. More targeted or exploratory approaches such as random forests [78] may prove useful in studying a wider range of scales, although identifying at which range remains a challenge [79, 80].

Since traps were not specifically deployed for the present study, the range of land-cover classes that could be studied without relying on extrapolation prevented the use of possibly more relevant land-cover classes that are important for mosquito habitats. For example, previous analyses using wetland cover as an explanatory variable led to extreme abundance values predicted in areas with important wetlands, although no traps were installed in such areas.

The large variance and the small range of the spatial field suggest that important variables for explaining mosquito abundance may operate on a very short scale, likely the local habitat variables surrounding traps [76]. However, we did not have access to such detailed habitat information, which likely prevented us from explaining a large part of the variations in mosquito abundances.

The number of traps varied greatly from year to year and trap locations changed across years. We initially sought to use a spatiotemporal model with autocorrelated weekly spatial components, but this led to convergence problems and very large uncertainties. Moreover, the variability in trap location across seasons and years coupled with the small range of the spatial component made predictions vary wildly across the study period. We expect a certain level of variation of abundances across years and seasons within the study area, however, it was unclear whether this variation was genuine or just an artefact of having trap locations changing across years and seasons. We thus opted for a simpler spatial model with which we could more easily focus on the main abundance pattern across the study area. Using a single seasonal effect common to each year facilitated the study of the mean seasonal pattern of mosquito abundance. Our recommendations for conducting mosquito abundance assessments are as follows:

1. Place the traps so they cover the range of possible values of the presumed most important habitat characteristics to avoid extrapolating mosquito abundance using predictor values not seen by the model.
2. Identify the most important local-scale habitat variables affecting mosquito abundance, which should facilitate the study of regional mosquito abundance patterns. Otherwise, strong local effects coupled with few trap locations may make the study of regional patterns of mosquito abundance more difficult. This will also likely help in reducing the large uncertainties associated with abundance predictions.
3. In situations where explained variances seem low, consider using a spatial component to better represent uncertainties in predicted abundances. Although adding a spatial component to a mosquito abundance model may make the prediction of abundance across space difficult, we feel it more adequately represents the uncertainty associated with predictions compared with a model without a spatial component. The importance of the spatial component in this study shows that despite relatively narrow uncertainties around predictions when considering only predictor variables, the uncertainty gets much larger when considering the spatial component, which indicates that abundance may vary wildly across space around what is predicted simply by predictors. Ignoring this uncertainty may give a false sense of confidence in the mapped pattern of mosquito abundances.

## Conclusions

The method presented in this paper to map abundances for various mosquito species in southern Quebec is a first step toward developing a practical tool that can be used in decision-making to identify areas of risk of exposure to infected mosquitoes. Indeed, no method had yet been developed in this study area that could be applied on a large scale and to four distinct species. The simplicity of the method used and the use of relatively generic land-cover and climate variables are encouraging and could easily be applied elsewhere. This type of model can also help to study the impact of climate and environmental change on the distribution of these mosquito species in time and space from a public health perspective. Indeed, temperature, precipitation and the presence of ‘urban’ areas are very important variables in our models and are undergoing profound changes: growing urban consumption of land, in tandem with an increase in mean temperatures and a change in precipitation patterns [81]. These three major effects can therefore be expected to significantly alter the landscape of mosquito abundances in the coming years and, consequently, the risk of exposure to mosquito-borne diseases.

## Electronic supplementary material

Below is the link to the electronic supplementary material.

Supplementary Material 1

Supplementary Material 2

Supplementary Material 3

Supplementary Material 4

## Data Availability

The data that support the findings of this study are available from GDG Environnement via the Public Health Agency of Canada, the Institut National de la santé publique du Québec and the Ministère de la santé et des services Sociaux du Québec but restrictions apply to the availability of these data, which were used under license for the current study, and so are not publicly available. Data are however available from the corresponding author upon reasonable request and with permission of GDG Environnement via the Public Health Agency of Canada, the Institut National de la santé publique du Québec and the Ministère de la santé et des services Sociaux du Québec.

## List of abbreviations

AAFC: Agriculture and Agri-Food Canada
Agri: Agricultural
AIC: Akaike Information Criterion
An: Annual
CDC: Center for Disease Control
CPR: *Culex pipiens-restuans* group
CQP: *Coquillettidia perturbans*
CSG: Californian serogroup
CVV: Cache Valley virus
DUC: Ducks Unlimited Canada
EEEV: Eastern Equine Encephalitis virus
GoC: Government of Canada
INSPQ: Institut national de santé publique du Québec
JCV: Jamestown Canyon virus
LCC: Land-cover class
MSSS: Ministère de la Santé et des Services sociaux
NHN: National Hydrographic Network
PHAC: Public Health Agency of Canada
SLEV: St. Louis encephalitis virus
SMG: *Ochlerotatus stimulans* group
SSHV: Snowshoe hare virus
VEX: *Aedes vexans*
VIF: Variance inflation factor
WNV: West Nile virus

## References

[1] Canada PH. A.o. West Nile virus and other mosquito-borne disease national surveillance report 2016—final- summary, P.H.A.o. Canada, Editor. 2017, Government of Canada: Ottawa, Canada. p. 13.

[2] Canada PHA. o. West Nile virus and other mosquito-borne disease national surveillance report 2015, P.H.A.o. Canada, Editor. 2017, Government of Canada: Ottawa, Canada. p. 14.

[3] Canada PH. A.o. West Nile virus and other mosquito-borne diseases in Canada, annual national surveillance report—2017, P.H.A.o. Canada, Editor. 2018, Government of Canada: Ottawa, Canada. p. 4.

[4] Canada PH. A.o. Mosquito-borne disease surveillance report—September 15 to 28, 2019 (week 40 & 41), P.H.A.o. Canada, Editor. 2019, Government of Canada: Ottawa, Canada. p. 4.

[5] Canada PHA. o. West Nile virus and other mosquito-borne diseases surveillance report: annual edition, 2018, P.H.A.o. Canada, Editor. 2020, Government of Canada: Ottawa, Canada. p. 11.

[6] Canada PH. A.o. Mosquito-borne disease surveillance report—September 27 to October 24, 2020 (week 40 to 43), P.H.A.o. Canada, Editor. 2020, Government of Canada: Ottawa, Canada. p. 5.

[7] Canada PH. A.o. Mosquito-borne diseases surveillance report - September 27 to October 24, 2021 (week 39 to 42), P.H.A.o. Canada, Editor. 2021, Government of Canada. p. 5.

[8] Québec. I.N.d.s.p.d. Virus du Nil occidental – tableau des cas humains - archives 2002–2020. Institut National de santé publique du Québec: Montréal, Québec, Canada; 2021.

[9] Québec IN. d.s.p.d. Surveillance des maladies d’intérêt transmises par les moustiques au Québec - Encéphalite équine de l’Est. 2020 [cited 2021 30 November 2021]; Available from: https://www.msss.gouv.qc.ca/professionnels/zoonoses/surveillance-des-maladies-d-interet-transmises-par-des-moustiques-au-quebec/encephalite-equine-de-l-est/.

[10] Québec IN. d.s.p.d. Surveillance des maladies d’intérêt transmises par les moustiques au Québec - Les virus du sérogroupe Californie. 2021 [cited 2021 30 November 2021]; Available from: https://www.msss.gouv.qc.ca/professionnels/zoonoses/surveillance-des-maladies-d-interet-transmises-par-des-moustiques-au-quebec/les-virus-du-serogroupe-californie/.

[11] Kotchi SO (2019). Using Earth observation images to inform risk assessment and mapping of climate change-related infectious diseases. Can Commun Dis Rep.

[12] Lebl K, Brugger K, Rubel F, Predicting. *Culex pipiens*/*restuans* population dynamics by interval lagged weather data. Parasites & Vectors, 2013. 6(1): p. 129. 10.1186/1756-3305-6-129 PMC3660179 23634763

[13] Rochlin I (2011). Predictive mapping of human risk for West Nile virus (WNV) based on environmental and socioeconomic factors. PLoS ONE.

[14] Yoo EH (2014). Site-specific prediction of West Nile virus mosquito abundance in Greater Toronto Area using generalized linear mixed models. Int J Geogr Inf Sci.

[15] Roiz D (2014). Climatic effects on mosquito abundance in Mediterranean wetlands. Parasites & Vectors.

[16] Sun H (2021). Spatio-temporal analysis of the main dengue vector populations in Singapore. Parasites & Vectors.

[17] Giordano BV, Turner KW, Hunter FF. Geospatial analysis and seasonal distribution of West Nile virus vectors (*Diptera: Culicidae*) in Southern Ontario, Canada. Int J Environ Res Public Health, 2018. 15(4). 10.3390/ijerph15040614 PMC5923656 29597256

[18] Cleckner HL, Allen TR, Bellows AS. Remote sensing and modeling of mosquito abundance and habitats in coastal Virginia, USA Remote Sensing, 2011. 3(12).

[19] El Adlouni S (2007). Effects of climate on West Nile virus transmission risk used for public health decision-making in Quebec. Int J Health Geogr.

[20] Jacob BJ (2009). Developing operational algorithms using linear and non-linear squares estimation in Python for the identification of *Culex pipiens* and *Culex restuans* in a mosquito abatement district (Cook County, Illinois, USA). Geospat Health.

[21] Wang X (2014). Clustering of the abundance of West Nile virus vector mosquitoes in Peel Region, Ontario, Canada. Environ Ecol Stat.

[22] Ripoche M (2019). Short-term forecasting of daily abundance of West Nile virus vectors *Culex pipiens-restuans* (*Diptera: Culicidae*) and *Aedes vexans* based on weather conditions in southern Québec (Canada). J Med Entomol.

[23] Wang J, Ogden N, Zhu H (2011). The impact of weather conditions on *Culex pipiens* and *Culex restuans* (*Diptera: Culicidae*) abundance: a case study in Peel Region. J Med Entomol.

[24] Lim A-Y (2021). Mosquito abundance in relation to extremely high temperatures in urban and rural areas of Incheon Metropolitan City, South Korea from 2015 to 2020: an observational study. Parasites & Vectors.

[25] Fornasiero D (2020). Inter-annual variability of the effects of intrinsic and extrinsic drivers affecting West Nile virus vector *Culex pipiens* population dynamics in northeastern Italy. Parasites & Vectors.

[26] Trawinski PR, Mackay DS (2010). Identification of environmental covariates of West Nile virus vector mosquito population abundance. Vector Borne Zoonotic Dis.

[27] Ha TV (2021). Spatial distribution of *Culex* mosquito abundance and associated risk factors in Hanoi, Vietnam. PLoS Negl Trop Dis.

[28] Chen L, Zhu H, Wang X (2019). Modeling spatiotemporal distribution of mosquitoes abundance with unobservable environmental factors. J Med Entomol.

[29] Reisen WK (2013). Ecology of West Nile virus in North America. Viruses.

[30] Andreadis TG, Anderson JF, Tirrell-Peck SJ (1998). Multiple isolations of eastern equine encephalitis and highlands J viruses from mosquitoes (*Diptera: Culicidae*) during a 1996 epizootic in southeastern Connecticut. J Med Entomol.

[31] Oliver J (2018). Twenty years of surveillance for eastern equine encephalitis virus in mosquitoes in New York State from 1993 to 2012. Parasites & Vectors.

[32] Gardner AM, Lampman RL, Muturi EJ (2014). Land use patterns and the risk of West Nile virus transmission in central Illinois. Vector-Borne and Zoonotic Diseases.

[33] Holmes CJ, Cáceres CE (2020). Predation differentially structures immature mosquito populations in stormwater ponds. Ecol Entomol.

[34] Cloutier CA, Fyles JW, Buddle CM. Diversity and community structure of mosquitoes (*Diptera: Culicidae*) in suburban, field, and forest habitats in Montréal, Québec, Canada. The Canadian Entomologist, 2021. 153(4): p. 393–411.

[35] Moua Y et al. Mapping the habitat suitability of West Nile virus vectors in southern Quebec and eastern Ontario, Canada, with species distribution modeling and satellite earth observation data. Remote Sens, 2021. 13(9).

[36] Dussault C (2018). Evaluating the impact of *Aedes japonicus* invasion on the mosquito community in the Greater Golden Horseshoe region (Ontario, Canada). PLoS ONE.

[37] Chuang TW (2012). Cross-correlation map analyses show weather variation influences on mosquito abundance patterns in Saginaw County, Michigan, 1989–2005. J Med Entomol.

[38] Turell MJ (2001). Potential north american vectors of West Nile virus. Ann N Y Acad Sci.

[39] Anderson JF et al. Arboviruses in North Dakota, 2003–2006. The American Society of Tropical Medicine and Hygiene, 2015. 92(2): p. 377–393. 10.4269/ajtmh.14-0291 PMC4347345 25487728

[40] Goddard LB (2002). Vector competence of California mosquitoes for West Nile virus. Emerg Infect Disease J.

[41] Main AJ et al. Arbovirus surveillance in Connecticut. II. California serogroup [*Aedes* species, insect vectors]. 1979. v. 39.

[42] Maire A, Aubin A. Les moustiques du Québec (*Diptera: Culicidae*) essai de synthèse écologique, in Groupe de recherche sur les insectes piqueurs, mémoires de la société entomologique du Québec. 1980, Université du Québec à Trois-Rivières, Québec, Canada:Québec, Canada. p. 107 pages.

[43] Rocheleau JP (2017). Characterizing environmental risk factors for West Nile virus in Quebec, Canada, using clinical data in humans and serology in pet dogs. Epidemiol Infect.

[44] Strickman D (1982). Stimuli affecting selection of oviposition sites by *Aedes vexans* (*Diptera*: *Culicidae*): light. J Med Entomol.

[45] Bosak PJ, Crans WJ (2002). The structure and function of the larval siphon and spiracular apparatus of *Coquillettidia perturbans*. J Am Mosq Control Assoc.

[46] Darold PB (2020). Phenology of *Coquillettidia perturbans* and *Culiseta melanura* (*Diptera*: *Culicidae*) in East-Central Georgia, USA: implications for the ecology of eastern equine encephalitis virus. J Entomol Sci.

[47] Bosak PJ, Reed LM, Crans WJ (2001). Habitat preference of host-seeking *Coquillettidia perturbans* (Walker) in relation to birds and eastern equine encephalomyelitis virus in New Jersey. J Vector Ecol.

[48] Andreadis TG (2008). Isolations of Jamestown Canyon virus (*Bunyaviridae*: *Orthobunyavirus*) from field-collected mosquitoes (*Diptera*: *Culicidae*) in Connecticut, USA: a ten-year analysis, 1997–2006. Vector Borne Zoonotic Dis.

[49] Maire A, Tessier C, Picard L (1980). Analyse éecologique des populations larvaires de moustiques (*Diptera*: *Culicidae*) des zones riveraines du fleuve Saint-Laurent. Québec Naturaliste canadien.

[50] Walker ED, Grayson MA, Edman JD (1993). Isolation of Jamestown Canyon and snowshoe hare viruses (California serogroup) from Aedes mosquitoes in western Massachusetts. J Am Mosq Control Assoc.

[51] Watts MJ (2021). The rise of West Nile virus in southern and southeastern Europe: a spatial-temporal analysis investigating the combined effects of climate, land use and economic changes. One health (Amsterdam Netherlands).

[52] Kottek MG, Jürgen G, Beck C, Rudolf B, Rubel F (2006). World Map of the Köppen-Geiger climate classification updated. Meteorol Z.

[53] Thornton MM, Shrestha R, Wei Y, Thornton PE, Kao S, Wilson BE. Daymet: daily surface weather data on a 1-km grid for North America, version 4. 2020; Available from: 10.3334/ORNLDAAC/1840.

[54] Hufkens K (2018). An integrated phenology modelling framework in r. Methods Ecol Evol.

[55] Wood SN (2011). Fast stable restricted maximum likelihood and marginal likelihood estimation of semiparametric generalized linear models. J Royal Stat Society: Ser B (Statistical Methodology).

[56] AAFC, Agriculture and Agri-Food Canada. ISO 19,131 AAFC Annual crop inventory—data product specifications—revision A. 2019. p. 26.

[57] Canada Go. Government of Canada. Land cover in government of Canada. Ottawa; 2019.

[58] Canada DU. Our work/impact area—Wetlands in Ducks Unlimited Canada, Conserving Canada’s wetlands. 2018.

[59] Canada. Go. Land cover in government of Canada. Ottawa; 2019.

[60] Geomatics PCIPCI. Geomatica. 2019; Available from: https://www.pcigeomatics.com/.

[61] Esri ArcGIS. Desktop: Release 10.6. Environmental Systems Research Institute. 2019; Available from: http://desktop.arcgis.com/en/.

[62] Rue H, Martino S, Chopin N (2009). Approximate bayesian inference for latent gaussian models by using integrated nested Laplace approximations. J Royal Stat Society: Ser B (Statistical Methodology).

[63] Lindgren F, Rue H, Lindström J (2011). An explicit link between Gaussian fields and Gaussian Markov random fields: the stochastic partial differential equation approach. J Royal Stat Society: Ser B (Statistical Methodology).

[64] Bakka H (2018). Spatial modeling with R-INLA: a review. WIRE Comput Stat.

[65] Musenge E, et al. 2010 in rural north east South Africa. Int J Appl Earth Obs Geoinf. 2013;22(100):86–98. Bayesian analysis of zero inflated spatiotemporal HIV/TB child mortality data through the INLA and SPDE approaches: applied to data observed between 1992. 10.1016/j.jag.2012.04.001 PMC3906611 24489526

[66] Myer MH, Campbell SR, Johnston JM (2017). Spatiotemporal modeling of ecological and sociological predictors of West Nile virus in Suffolk County, NY, mosquitoes. Ecosphere.

[67] Myer MH, Johnston JM (2019). Spatiotemporal bayesian modeling of West Nile virus: identifying risk of infection in mosquitoes with local-scale predictors. Sci Total Environ.

[68] Fuglstad G-A (2019). Constructing priors that penalize the complexity of gaussian random fields. J Am Stat Assoc.

[69] Simpson D (2017). Penalising model component complexity: a principled, practical approach to constructing priors. Stat Sci.

[70] Fox J, Weisberg S, An. R companion to applied regression, Third edition, ed. Sage. 2019, Thousands Oaks (CA).

[71] Van Niekerk J, Krainski ET, Rustand D, Rue H. A new avenue for Bayesian inference with INLA. arXiv preprint, 2022: p. arXiv:2204.06797.

[72] Gelman A, Hwang J, Vehtari A (2014). Understanding predictive information criteria for bayesian models. Stat Comput.

[73] Hooten MB, Hobbs NT (2015). A guide to bayesian model selection for ecologists. Ecol Monogr.

[74] Hartig F. DHARMa: residual diagnostics for hierarchical (multi-level/mixed) regression models. R package version 0.4.4. 2021; Available from: https://CRAN.R-project.org/package=DHARMa.

[75] Dunn PK, Smyth GK (1996). Randomized quantile residuals. J Comput Graphical Stat.

[76] Laporta GZ, Sallum MAM. Coexistence mechanisms at multiple scales in mosquito assemblages. BMC Ecol, 2014. 14(1). 10.1186/s12898-014-0030-8 PMC4247778 25384802

[77] Hopkins MC et al. Influence of forest disturbance on La Crosse virus risk in southwestern Virginia. Insects, 2020. 11(1). 10.3390/insects11010028 PMC7022478 31905866

[78] Bradter U (2013). Identifying appropriate spatial scales of predictors in species distribution models with the random forest algorithm. Methods Ecol Evol.

[79] Jackson HB, Fahrig L (2015). Are ecologists conducting research at the optimal scale?. Glob Ecol Biogeogr.

[80] Stuber EF, Fontaine JJ (2019). How characteristic is the species characteristic selection scale?. Glob Ecol Biogeogr.

[81] Ogden NH, Gachon P. Climate change and infectious diseases: what can we expect? Can Commun Dis Rep, 2019. 45(4). 10.14745/ccdr.v45i04a01 PMC6587697 31285696

