# Supplementary figures and images for: Mapping the abundance of endemic mosquito-borne diseases vectors in southern Quebec
Source: BMC Public Health. 2023 May 22;23:924. doi: 10.1186/s12889-023-15773-x (PMC10204333; doi:10.1186/s12889-023-15773-x)

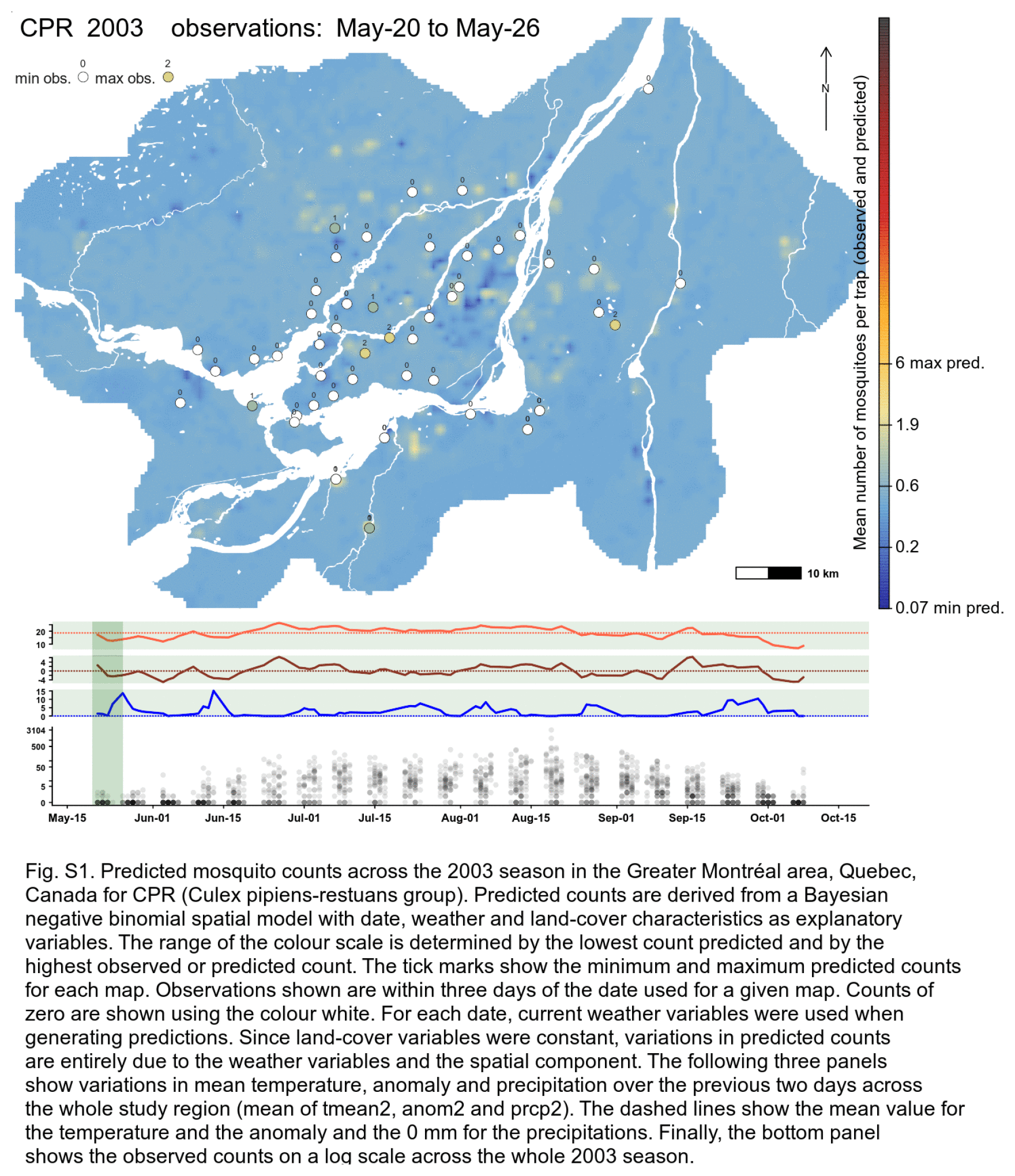

Supplement: Supplementary file 1 — Supplementary Material 1 [file 12889_2023_15773_MOESM1_ESM.gif]

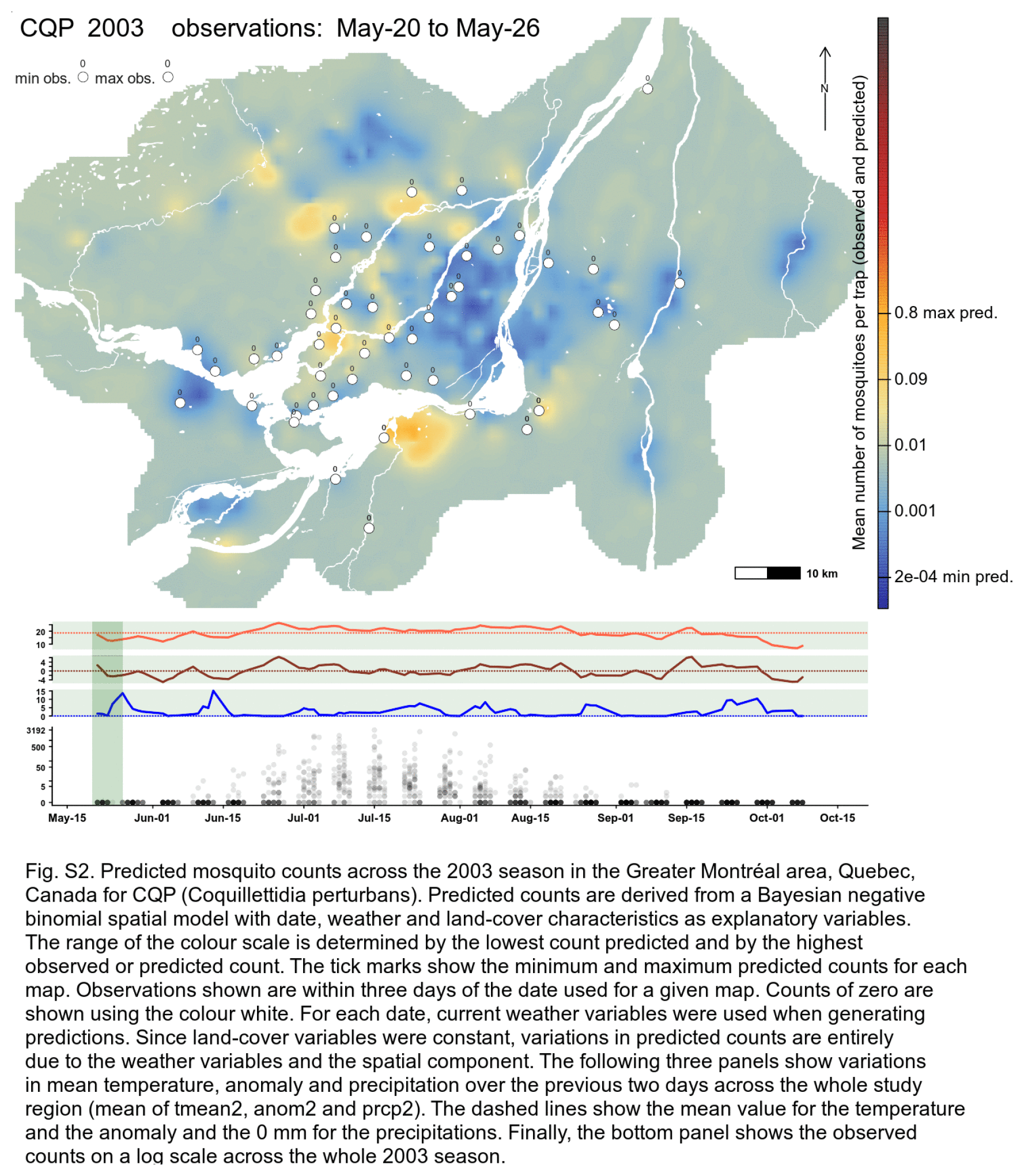

Supplement: Supplementary file 2 — Supplementary Material 2 [file 12889_2023_15773_MOESM2_ESM.gif]

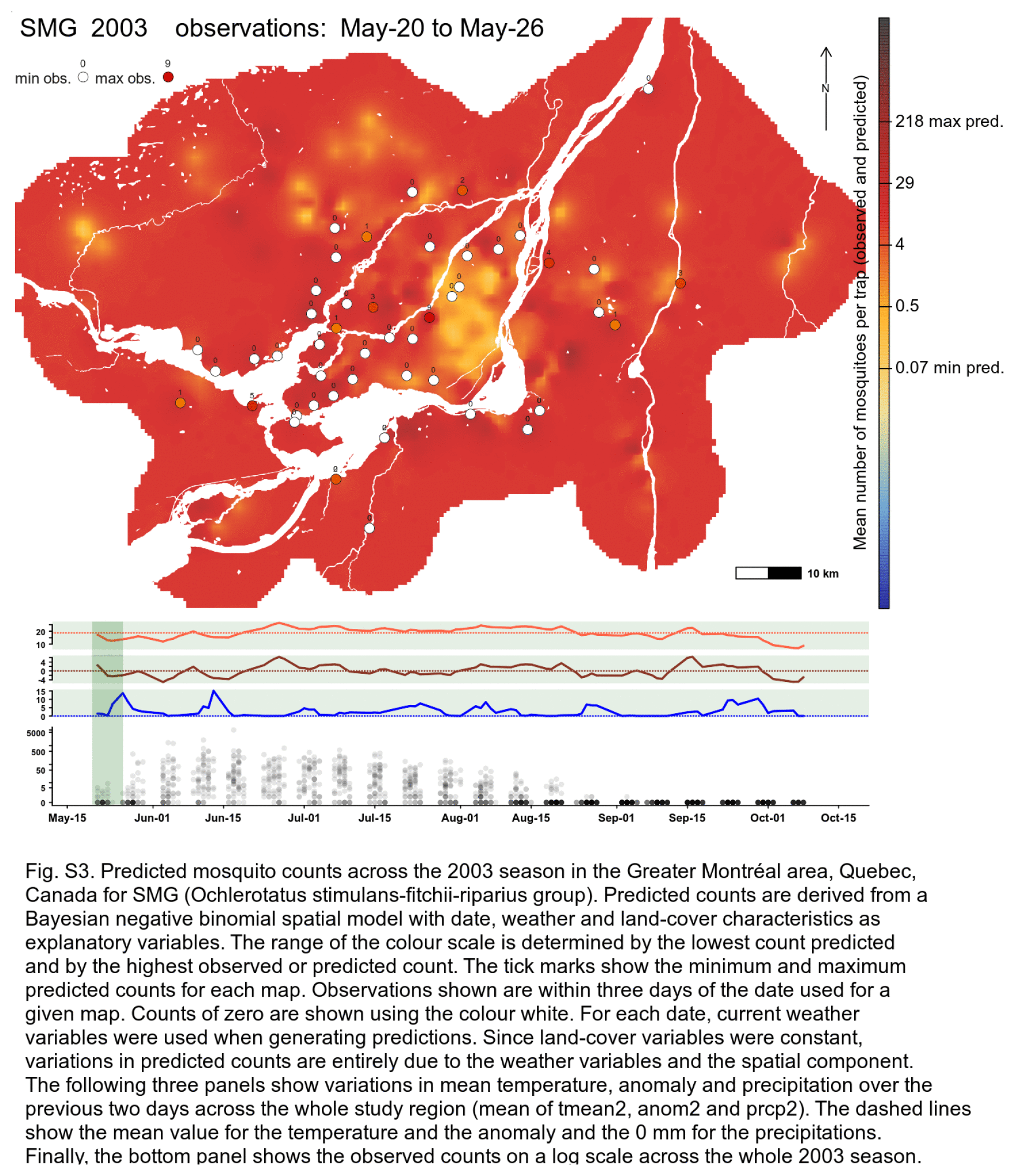

Supplement: Supplementary file 3 — Supplementary Material 3 [file 12889_2023_15773_MOESM3_ESM.gif]

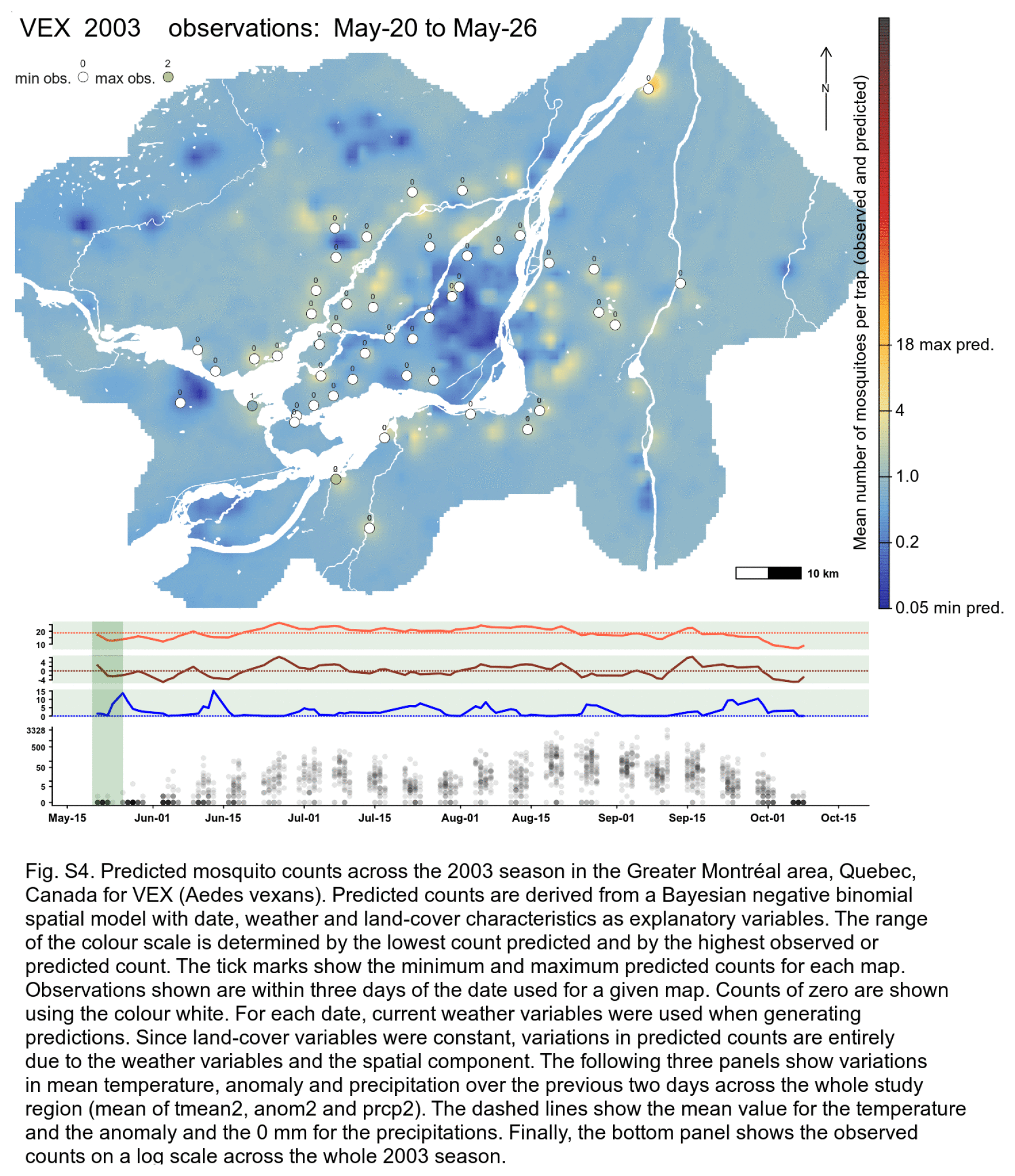

Supplement: Supplementary file 4 — Supplementary Material 4 [file 12889_2023_15773_MOESM4_ESM.gif]
